# Genetic polymorphisms and their association with brain and behavioural measures in heterogeneous stock mice

**DOI:** 10.1038/srep41204

**Published:** 2017-02-01

**Authors:** Magdalena Janecka, Sarah J. Marzi, Michael J. Parsons, Lin Liu, Jose L. Paya-Cano, Rebecca G. Smith, Cathy Fernandes, Leonard C. Schalkwyk

**Affiliations:** 1 MRC Social, Genetic and Developmental Psychiatry Centre, Institute of Psychiatry, Psychology and Neuroscience, King’s College London, London, UK; 2 Mammalian Genetics Unit, MRC Harwell, Oxfordshire, UK; 3 School of Biological Sciences, University of Essex, Colchester, UK

## Abstract

Although the search for quantitative trait loci for behaviour remains a considerable challenge, the complicated genetic architecture of quantitative traits is beginning to be understood. The current project utilised heterogeneous stock (HS) male mice (n = 580) to investigate the genetic basis for brain weights, activity, anxiety and cognitive phenotypes. We identified 126 single nucleotide polymorphisms (SNPs) in genes involved in regulation of neurotransmitter systems, nerve growth/death and gene expression, and subsequently investigated their associations with changes in behaviour and/or brain weights in our sample. We found significant associations between four SNP-phenotype pairs, after controlling for multiple testing. Specificity protein 2 (*Sp2,* rs3708840), tryptophan hydroxylase 1 (*Tph1,* rs262731280) and serotonin receptor 3A (*Htr3a,* rs50670893) were associated with activity/anxiety behaviours, and microtubule-associated protein 2 *(Map2,* rs13475902) was associated with cognitive performance. All these genes except for *Tph1* were expressed in the brain above the array median, and remained significantly associated with relevant behaviours after controlling for the family structure. Additionally, we found evidence for a correlation between *Htr3a* expression and activity. We discuss our findings in the light of the advantages and limitations of currently available mouse genetic tools, suggesting further directions for association studies in rodents.

Quantitative traits (QT) are phenotypes that vary continuously in the population, likely due to their polygenic background. Examples of QTs include anthropometric measures (height), behaviour (neuroticism) and physiological profiles (alcohol metabolism). Genome-wide research in humans and animals has long tried to identify QT loci (QTLs), chromosomal regions where allelic variation is associated with a measurable change in those traits^1^,^2^. Nevertheless, this remains a challenge due to the highly complicated genetic architecture of QTs, with phenomena like pleiotropy, polygenicity, gene-gene and gene-environment interactions most likely involved.

Investigating the genetic underpinnings of behaviour and psychiatric disorders is further complicated by the phenotypic complexity of these measures. The currently prevailing view is that most of them are multi-faceted constructs, fractionable into a number of molecular and behavioural processes (endophenotypes)^3^,^4^. Importantly, different disorders often involve overlapping endophenotypes – a phenomenon, which explains common genetic variation between them, rendering the search for disorder-specific loci problematic. For this reason, investigating the endophenotypes’, rather than the disorders’ QTLs may be more attainable.

The relative simplicity of these measures allows for employing model organisms in attempts to understand the genetic basis of behaviour. Mouse models have been highly instrumental in behavioural genetic research, offering scientists a variety of different tools (e.g. knock-out models, congenic strains, outbred populations) that allow for fine dissection of the complex psychiatric disease phenotypes. In our study we used heterogeneous stock (HS) mice, an outbred population derived from eight inbred strains: A/J, AKR, BALB/c, C3H/2, C57BL, DBA/2, Is/Bi and RIII^5^ that have been intercrossed for over 70 generations, with pseudo-random breeding protocols to achieve high levels of recombination^6^. The HS mice have a number of advantages in genetic research, including some the highest resolution mapping of QTLs (of up to 1Mb^7^) offered by mouse genetic models^8^,^9^.

Using HS mice we aimed to identify the genetic underpinnings of behavioural domains affected in a number of psychiatric disorders, namely anxiety, activity and cognitive phenotypes. We investigated the association between behavioural tests that provide indices of these functions and a series of single nucleotide polymorphisms (SNPs) in candidate genes involved in major neurotransmitter systems (glutamate (Glu), serotonin (5HT), dopamine (DA), acetylcholine (Ach), GABA, noradrenaline (NA), and the endocannabinoid system), neuronal growth/death and gene expression (Table S1). We also tested if genetic polymorphisms in these loci could predict variance in the brain measures (cerebellum, hippocampus, whole brain weights) of these animals. Finally, all significant SNP-phenotype associations were supplemented with expression data analysis from the same individuals. Although the list of genes we investigated was by no means exhaustive, nominating a more limited number of loci from across a range of biological pathways offered us a better scope to identify the molecular background of the phenotypes we were interested in than narrower, more in-depth search.

Together with our findings, we discuss the validity of HS as a tool for genetic research, and suggest further directions for genetic association studies in mice.

## Results

### Allele frequency

There were 126 SNPs in our original set of loci within genes of potential behavioural relevance (synaptic transmission, nerve growth/death, gene expression). Of these, 94 SNPs were in Hardy-Weinberg equilibrium (HWE) with a p > 0.05 cut-off, and further 10 reasonably close to it, warranting their inclusion in the analyses. Within the final sample of 104 SNPs, a substantial number of SNPs had very low minor allele frequencies (MAF < 0.125). Table S2 presents the frequencies of minor allele homozygotes and heterozygotes for all the SNPs genotyped.

Interrogation of the mouse SNP database published by the Wellcome Trust Sanger Consortium^10^ showed that in the HS founders, there were 2,724 SNPs annotated as exonic, out of which 317 were highly polymorphic between the HS founding strains.

### Data properties

Descriptive statistics for the behavioural and brain data are presented in Tables S3 and S4, respectively. The Shapiro-Wilk tests indicated that only measures of mean speed upon transfer to home cage were normally distributed in the HS sample (W = 0.992, p = 0.061; for other behavioural measures: 0.468 < W < 0.985; 2.2E-16 < p < 2.5E-5; brain measures: 0.602 < W < 0.978; 2.2E-16 < p < 6.0E-6). However, visual inspection suggested that the distribution in most of the measures approximated normal; the exceptions were the measures representing latencies in the Puzzle Box and anxiety indices, where the distributions were censored (variables marked in the Table S3 with an (*)).

Spearman rank correlation confirmed that most measures within each behavioural test, except for the Morris Water Maze (MWM) measures, were significantly correlated with each other (p_mean_ = 0.040, p_MWM_ = 0.495), and that this correlation was moderate (**ρ**_mean_ = 0.323, **ρ**_MWM_ = 0.032; calculations performed using absolute rho values). Given the degree of correlation within, but not between tests (**ρ**_mean_ = 0.134), the number of behavioural tests, rather than total measures was used when correcting for multiple testing. Due to the lack of correlation between MWM measures, they were treated as separate tests in the False Discovery Rate protocol used to control for multiple testing.

All brain weights were shown to be significantly and positively correlated with each other and body weight (0.103 < **ρ** < 0.421). The exception was hippocampal weight, which was not significantly correlated with body weight (Table S5, Figure S3).

Analysis of variance revealed that most of the measures, of both brain and behaviour, showed significant batch effects (F_mean_ = 20.712, p_mean_ = 0.159); this justified our approach of regressing the batch effects out of all our analyses.

### Effects of genetic polymorphisms on behaviour and brain measures

**Figure 1:**
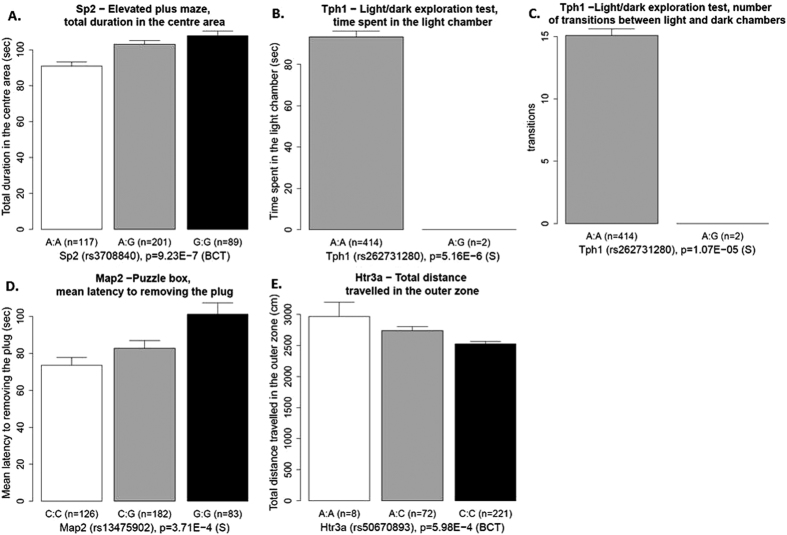
SNP-behaviour associations that remained significant after multiple testing correction. Each graph presents the mean values for the given phenotype, broken down by genotype, with error bars showing the SE. The panels represent the associations between (**A**) Sp2 (rs3708840) and total duration in the centre area of the elevated plus maze, (**B**) Tph1 (rs262731280) and time spent in the light chamber during the Light/dark exploration test, (**C**) Tph1 (rs262731280) and number of transitions between light and dark chambers during the Light/dark exploration test, (**D**) Map2 (rs13475902) and mean latency to remove the plug in the Puzzle box test, and (**E**) Htr3a (rs50670893) and total distance travelled in the outer zone of the open field. SE. The p-values represent the significance level of the association, also indicating which model was used in the test (BCT – linear model with Box-Cox transformed values; S – Cox proportional hazards survival model).

Out of all of the SNP-behaviour pairs tested, only associations between (i) *Sp2* (rs3708840) and total duration in the centre area of the elevated plus maze, (ii, iii) *Tph1* (rs262731280) and number of transitions between light and dark chambers, and time spent in the light chamber remained significant after multiple-testing correction at q = 0.01. Additionally, (iv) *Map2* (rs13475902) and mean latency to removing the plug in the puzzle box, and (v) *Htr3a* (rs50670893) and peripheral activity in the open field were significant at q = 0.05 (Fig. 1, Table 1). After also controlling for the family structure, all but (ii) were significant at q = 0.05, and all but (ii) and (iii) at q = 0.01. All SNP-brain measure associations were below the required threshold.

The top 100 SNP-phenotype associations (p < 0.02) analysed for the possibility of gene-behavioural domain clusters revealed significant enrichment (Chi^2^(14) = 37.693, p = 6.0E-4; Table 2) in some functional groups (full list of these associations in Table S6).

In the Northport Stock (Wellcome Trust Centre for Human Genetics, http://mus.well.ox.ac.uk/mouse/HS/), there was a QTL for distance travelled in the closed arms of the elevated plus maze at ch11: 92,322,571–96,137,510, very close to our significant *Sp2* region (ch11: 96,953,340–96,982,959). There was also a region associated with activity in the open field arena on the *Htr3a* gene, as well as further upstream on chromosome 9. The *Tph1* region did not harbour any anxiety-related QTLs, and there were no cognition-related phenotypes available for the Northport Stock, precluding validation of our *Map2* results in an independent dataset.

### Associations between gene expression and behaviour

Gene expression profiles of the cortex tissue were performed for a subset (n = 265) of mice. For the genes containing polymorphisms associated with behavioural measures, *Sp2* (6.131), *Map2* (11.389) and *Htr3a* (7.323) showed expression above the array median (5.996), while *Tph1* (3.900) expression was below the median. Testing for associations between gene expression and the corresponding behavioural measures revealed that only *Htr3a* expression was significantly associated with peripheral activity in the open field (*p* = 0.011, Table S7), where higher *Htr3a* expression was linked to a shorter distance travelled in the outer zone of the open field (Fig. S4). Association between *Map2* expression and plug removal was also near-significant (p = 0.077, Table S7), with higher gene expression predictive of shorter latency to perform the task. Given expression analyses was performed only on the SNP-phenotype pairs that remained significant in the genetic association testing after the FDR correction (5 pairs), p-values presented in this section were not corrected for multiple testing.

## Discussion

### Genetic effects on behaviour and brain measures in HS mice

**Figure 2:**
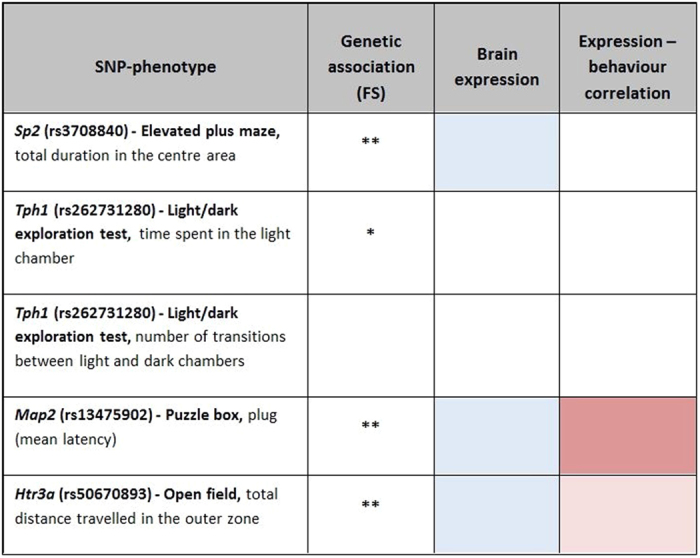
Summary of the findings for the top 5 SNP-phenotype association. The figure represents degree of significance, after controlling for family structure (FS) effects at q = 0.01 (**) or q = 0.05, gene expression detectable in the brain above the array median and correlation between expression levels and behaviour (dark grey for significant at p < 0.05; light grey for near-significant at p < 0.1).

Figure 2 shows the full summary of our results from the genetic association and brain expression analyses. We observed associations between loci on *Sp2* (rs3708840) and *Tph1* (rs262731280) and anxiety phenotype measures, which remained significant after multiple testing correction at q = 0.01. In both cases the polymorphism was associated with a missense mutation predicting change of amino acids with different hydropathy indices, likely to affect functioning of the protein they form, and its interactions with other molecules. At a lower significance level (q = 0.05) we also noted associations between a SNP on *Map2* (rs13475902) and latency to remove a cardboard plug, which blocked the underpass in the puzzle box test, as well as between a SNP on *Htr3a* (rs50670893) and distance travelled in the outer zone of the open field. Expression analysis revealed that all of these genes, with the exception of *Tph1*, were detected in the brain (expression levels above the array median). Expression levels of *Htr3a* were also significantly associated with the open field behaviour, with an indication of similar effects between *Map2* expression and latency to remove the plug in the puzzle box.

Comparison of our results with the Wellcome Trust resource^11^ provided support for some (*Sp2, Htr3a*), but not all of these associations. As mentioned above, this could be expected given only partially overlapping sets of inbred strain founders of the HS Northport and Boulder stocks. The other important difference between ours and Wellcome Trust dataset was the number of mice geno-/phenotyped – although Valdar *et al*.^11^ analysed higher number of individuals, these included multiple mice from the same litters, likely resulting in higher family structure (and lower genetic variance) than in our set. The level of genetic and phenotypic detail provided by these datasets also differs – we conducted more behavioural assessments than available in the Wellcome Trust resource; on the other hand, the latter dataset offers genome-wide genetic information, in contrast to candidate-gene analysis performed in our study. On the whole, both of these datasets are associated with several strengths and limitations, and convergence of our and Northport findings is encouraging.

Finally, analysis of the most significant associations further revealed a presence of functional clusters, with SNPs on genes involved in particular neuronal functions/neurotransmitter pathways being more likely involved in phenotypes recorded in our sample than what would be expected by chance. All results are discussed in more detail below.

### Functional clusters

Analysis of the 100 most significant associations among our candidate genes revealed an enrichment of genes involved in particular biological pathways within distinct behavioural domains. Within these top-scoring associations, there was an enrichment of loci on genes implicated in 5HT, Ach and glutamate regulation and activity/anxiety phenotypes. Also, SNPs associated with cognitive performance were located mainly on genes involved in GABAergic regulation. Finally, the majority of SNPs on genes involved in neuronal growth/death found in these top-scoring associations were involved in cognitive phenotypes (this last finding warrants caution due to very low numbers of SNPs in this group (5, out of which 4 were associated with cognitive measures). Extensive literature supports the biological plausibility of all of these SNP-phenotype clusters^12^,^13^,^14^, and these observations may reflect the underlying polygenicity of the traits we investigated. None of the SNP associations with brain measures remained significant after multiple testing correction (see the discussion below). However, clustering of the strongest associations with loci on GABA and 5HT genes – both involved in early neurodevelopment – warrants further investigation.

### Genetic associations with behaviour

#### Sp2-elevated plus maze

The most significant SNP-behaviour association was between a SNP on *Sp2* (rs3708840; missense mutation predicting threonine to isoleucine change, T166I) and an anxiety-related measure, time spent in the central area of the elevated plus maze test. In our sample, carriers of at least one minor allele spent significantly more time in the central area of the maze, an area thought to be associated with decision-making/avoidance of a potentially threatening environment^15^ - suggesting increased anxiety propensity in these individuals. *Sp2* belongs to the family of specificity proteins (Sps) acting as transcription factors. The gene was shown to be crucial in early murine development^16^, affecting both cell proliferation and differentiation^17^,^18^, but not much is known about its role in the context of behaviour. Our results from the expression analysis showed that the gene is brain-expressed in our sample, suggesting its role in behaviour is highly plausible. Nevertheless, we did not observe an association between its expression levels and behaviour, indicating that the gene’s polymorphism associated with anxiety phenotypes is likely to operate through altering properties of the protein, rather than any dose-dependent effects. Consistently with this explanation, in humans, SNPs in the SP2 region reside in the regulatory sequence (rs370243877, rs796052870), although the consequences of these polymorphisms are yet to be characterised.

Comparing our results with the Wellcome Trust data on Northport Stock HS mice provided further support for the importance of the *Sp2* region in anxiety regulation. We found that a locus (ch11: 92,322,571–96,137,510) very close to *Sp2* (ch11: 96,953,340–96,982,959) was associated with the distance travelled in the closed arms of the elevated plus maze in the Wellcome Trust resource. Although we did not examine this measure in our study, frequency of entry into closed arms in our stock was significantly correlated with time spent in the central area of the elevated plus maze test (the phenotype associated with *Sp2*). Furthermore, both the central zone and the closed arms are considered “protected areas” of the maze, therefore parameters recorded in these zones are likely regulated by the same behavioural components^15^. Therefore, it is possible that the measures associated with a QTL on chromosome 11 in our sample and Northport stock tap into the same phenotype, regulated by the *Sp2* region in the mouse genome. Converging evidence from the association analyses in these two datasets, properties of the SNP analysed by us and brain expression of *Sp2* all suggest that the gene can play an important role in anxiety regulation. Further verification of these effects could eventually be performed by analysing the knock-out phenotype, however, there is no data in the International Mouse Phenotyping Consortium database (IMPC; http://www.mousephenotype.org/) at the time of writing this manuscript).

#### Tph1-light dark box

Two anxiety-related phenotypes measured in the light-dark exploration test showed significant association with a locus on *Tph1 (*rs262731280; missense mutation predicting tyrosine to cysteine change, Y206C), encoding tryptophan hydroxylase, a rate-limiting enzyme involved in 5HT synthesis. Minor allele carriers were more anxious, spending significantly less time in the light chamber, and making fewer transitions between light and dark chambers. Nevertheless, our findings warrant caution due to very low number of heterozygotes (n = 2), and lack of animals homozygous for the minor allele. Furthermore, the levels of expression were below the array median, and they did not associate with behaviour. Finally, analysis controlling for the relatedness in our sample suggested that the significant effects were likely driven by the family structure.

Indeed, the role of Tph1 in behaviour remains controversial. Traditionally it has been considered to be expressed primarily in the periphery^19^ and normal 5HT levels were recorded in brains of Tph1−/− mice19. However, some more recent evidence from rodents and humans suggests that the gene is expressed in the raphe nuclei^20^,^21^, although not as robustly as the Tph2 isoform. The Wellcome Trust HS resource does not contain measures from the light dark exploration test, and none of the regions reported to associate with elevated plus maze performance (also indexing anxiety) in Northport Stock were on chromosome 7, where Tph1 is located.

#### Htr3a – open field

The SNP in *Htr3a* investigated in our study (rs50670893) predicts a synonymous change in the protein-coding section of the gene. In our sample, carriers of the minor T allele displayed higher locomotor activity in the periphery of the open field arena, and this effect was strongest in the homozygous individuals. We also observed significant association between levels of *Htr3a* expression in the brain and behaviour – suggesting the mechanisms driving the SNP-phenotype association are likely expression related.

*Htr3a* (serotonin receptor 3 A) has an established role in behaviour regulation^22^, having been linked with harm avoidance^23^, schizophrenia^24^ (and its treatment response^25^) and anxiety disorders^26^,^27^,^28^, alongside other neuropsychiatric traits (see review^29^). Although distance travelled in the outer zone of the open field is usually considered to index a locomotor activity rather than anxiety phenotype, given that the test is carried out in a novel arena, this measure likely also reflects the latter behavioural domain. In the Northport Stock *Htr3a* region was associated with activity in the open field (data for the outer zone alone is not available), with multiple additional association peaks in its wider genomic context on chromosome 9, validating our results from the Boulder stock. Similarly as for *Sp2*, the phenotypic consequences of *Htr3a* knock-out have not been described to date, although our results definitely warrant further interest in the role of *Htr3a* in behavioural regulation.

#### Map2-puzzle box

Finally, a *Map2* polymorphism we investigated was a non-synonymous mutation predicting change from glycine to alanine (rs13475902, G987A), both small aliphatic amino acids. The polymorphism within the mRNA coding region could contribute to alterations in the gene expression, although, to the best of our knowledge, experimental evidence for the role of this SNP in this is still lacking.

In our sample the SNP was associated with latency to remove a plug in a puzzle box test, a problem-solving test (mice are required to complete consecutive puzzles, of which removal of the plug is the most difficult one). Carriers of the minor G allele spent significantly more time before successfully removing the plug, suggesting lower cognitive ability in these animals.

Although the association between expression levels and behaviour was just above our significance cut-off (p = 0.08), this suggests *Map2* may be involved in the cognitive processes captured by the puzzle box test. Given that the gene is expressed ubiquitously in mouse brain (Fig. S6, Image credit: Allen Institute^30^), our expression analysis might have lacked spatial specificity to uncover the relationship between the expression levels and function. Individual mice with higher expression of the gene showed improved performance, as indexed by lower latency to remove the plug. These results are consistent with the literature, with previous studies showing a role of *Map2* in cognitive performance in rats^31^,^32^, with reduced expression of the protein in individuals showing cognitive impairment. In humans, reduced expression was observed in hippocampi of schizophrenia patients^33^, a disorder that in rodents is, together with other tests, modelled using the puzzle box. Although we could not cross-validate our results in the Northport stock (due to the absence of cognitive measures for these individuals) nor in the IMPC knock-out resource (no phenotype available) previous evidence linking *Map2* with cognitive function provides strong encouragement for further investigation of the role of this gene in behaviour.

### Genetic associations with brain measures

None of the associations between our SNPs and brain measures remained significant after correcting for multiple testing. This does not imply the genetic variation we considered has no effect on brain function, as brain weights provide only fairly crude assessment of neural processes. Differences in connectivity patterns or synaptic efficiency, which could affect the behaviour in our sample, could not be assessed by the measures we took. Also, some structures that might have been crucial for the behaviours under analysis (e.g. amygdala in anxiety, frontal cortex in cognitive tests) were not included in the current study. Finally, although all brain dissections were carried out by the same individual, a degree of variation in the dissection procedure likely have remained.

Nevertheless, of note is the enrichment of SNPs involved in 5HT and GABA signalling among our top hits. Both of these neurotransmitters play prominent roles in early neurodevelopment, regulating processes like cell proliferation, neural outgrowth and apoptosis^34^,^35^. Interestingly, hippocampal weights were not correlated with body weight, unlike the total brain and cerebellar weights. Although this may result partly from the noise associated with the dissection, it has been suggested that hippocampal weight may be regulated by a unique set of genes compared to body weight^36^.

### Genetic polymorphism in HS mice

There is a considerable scope for genetic variation in the HS stock, as revealed by our interrogation of the mouse SNP database published by the Wellcome Trust Sanger Consortium^10^, which includes information on over 65 million SNPs in 18 inbred strains. In the HS founders, there were 2,724 SNPs annotated as exonic, with 317 (11.64%) out of them being highly polymorphic (allelic ratio of at least 4:6 in the founder/related strains – see the Online search section of Methods; due to divergence of certain lines used in the Boulder stock we looked at 10, rather than 8 strains). Given that there were altogether 553,545 polymorphic loci among these strains, using estimates from the exom presented above, one would expect 64,433 (11.64%) highly polymorphic SNPs of diverse genomic locations; this estimate will be affected by certain loci displaying preferential contribution of one of the founder strains^37^. This suggests that there is a large scope for maintaining genetic diversity in the HS over generations, even without taking into account *de novo* mutations that are likely to appear within the stock over time.

Although it is predicted that due to a genetic drift some of this variance will disappear over generations and alleles will become fixed in the population^38^, the rate of that has been shown to be relatively slow. Over > 50 generations in the Northport HS, 7.8% polymorphic alleles underwent fixation^6^. Those, however, probably included mainly the alleles that, although polymorphic, had low minor allele frequency, as fixation would act preferentially on those^39^. Eventually, highly polymorphic alleles would become fixed as well, but the time before that occurs is going to be substantially longer.

Nevertheless, some of the interrogated SNPs in our sample turned out to be out of HWE in our sample. This was not due to technical errors with the genotyping but due to our sample population as analysis by Mott and co-workers^40^ suggests that minor allele frequency in HS stocks is generally a limiting factor.

### Limitations and future directions

Our study successfully uncovered several gene-behaviour associations, and demonstrated a significant enrichment in associations between distinct behavioural domains and loci on genes that were shown to be biologically relevant in these phenotypes. Nevertheless it must be borne in mind that all of the associations presented in this paper are statistical inferences, rather than experimental evidence of biology.

With regard to family structure, we used a design with a single offspring per pairing that, combined with the pseudorandom mating scheme largely avoided its effects. In practice, in a few cases up to 3 siblings from a litter were used, which could possibly inflate the strength of associations reported in our paper. We implemented the best available correction for this issue, including the family identity as a random effect in the model.

We are also aware of the limitations stemming from the candidate gene approach, differences between genetic architecture in mice and humans, more extensive linkage disequilibrium in mice then humans^41^, as well as inherently difficult translation of behavioural findings from mouse models to humans. Due to polygenicity of most complex traits, most QTLs are likely to be of only very small phenotypic effect, and our study design had limited power to uncover such loci.

Finally, several methodological factors might have limited our scope in uncovering genetic associations with brain and behaviour measures in mice. A large number of hypotheses were tested, which is particularly problematic in the context of low minor allele homozygotes at many of the investigated loci. In future research the latter problem could be addressed by mating the HS mice with inbreds homozygous for the minor allele at the SNP under investigation; such an approach would inflate the number of minor allele homozygotes, resulting in higher statistical power.

## Materials and Methods

### Animals

HS mice were obtained from the Institute of Behavioral Genetics (IBG) at the University of Colorado, Boulder (USA) and shipped to the UK at the age of 8 weeks. The mice were tested on a battery of behavioural tasks (see Table 3 and ref. 42) and culled aged 5 months. Given the large sample size, mice were tested in 9 batches of approximately 80–100 mice per batch. All animal work was licensed under the Animals (Scientific Procedures) Act 1986, reviewed and approved by the ethical review panel of the Institute of Psychiatry and the Home Office inspectorate, and was in accordance with the European Communities Council Directive of 24 November 1986 (86/609/EEC). Project licence reference PPL 70/6342.

### Sample collection and processing

#### Brain weights

At culling, brain samples were dissected and weighed, as described previously^43^. Briefly, immediately after cervical dissection, the whole brain (including olfactory bulbs and brainstem) was carefully removed from the skull. Whole brains were placed ventral side down on a sterile petri dish containing ice-cold artificial cerebrospinal fluid (aCSF: 124 mM NaCl, 3 mM KCl, 1.25 mM NaH_2_PO_4_, 2 mM CaCl_2_.2H_2_O, 2 mM MgCl_2_.6H_2_O, Sigma, UK) over ice and dissected into a number of distinct brain regions. The brains were transected at the caudal margin of the cerebellum and the olfactory bulbs dissected out by cutting the fissura rhinalis using a scalpel blade. Wet cerebri (whole brain minus cerebellum and olfactory bulbs) were blotted on filter paper and immediately weighed to the nearest 0.1 mg. The right and left hippocampus and cerebellum were dissected on the petri dish over ice, blotted on filter paper and weighed. The following brain weights were collected: total wet brain weight, hippocampus (left and right combined) wet weight, and cerebellum wet weight.

#### DNA extraction and genotyping

DNA for genotyping was extracted from the spleen using a standard phenol/chloroform protocol^44^. In some cases of an animal’s unexpected death, spleen samples were not available and DNA was extracted from a tail sample. Following the extraction, DNA was diluted 1/50 in Tris-EDTA buffer and stored at 4 °C. Genotyping was performed using primer extension methods (SNuPe for the in-house genotyping on the MegaBASE, and Amplifluor for KBiosciences genotyping).

#### RNA extraction and gene expression

RNA for expression analysis was extracted from cortex tissue using TRIzol reagent (Invitrogen, Paisley, UK). Data from 580 adult male HS mice (generations 66–72) was used for the association testing, with expression data available for 265 out of these. Briefly, brains were removed from the skull and placed dorsal side down on a wetted filter paper on a petri dish kept on ice. The cerebral halves were opened out from the midline, after cutting through the corpus callosum. Approximately 3 mm^3^ of tissue was cut from the anterior part of the frontal lobes (from bregma 2.46 mm to 1.34 mm^45^), mainly containing the medial prefrontal cortex including some prelimbic cortex, infralimbic cortex, cingulate cortex and motor cortex^46^. Expression profiles of the cortex tissue were determined using the Affymetrix GeneChip^®^ Mouse Exon 1.0 ST Array (Santa Clara, CA, USA).

### SNP selection

We investigated 126 SNPs within 78 candidate genes – see Table S1 for the full list; the locations of the SNPs were determined using Ensembl Mus musculus version 78.38 (GRCm38 p.3; http://www.ncbi.nlm.nih.gov/projects/genome/assembly/grc/mouse/), and SNP rs numbers come from the dbSNP database (http://www.ncbi.nlm.nih.gov/projects/SNP/). In this SNP set we included loci involved in major neurotransmitter pathways (66 genes, 114 SNPs), gene expression regulation (5 genes, 5 SNPs), and neuronal growth/death (7 genes, 7 SNPs). It was not feasible to comprehensively assess all loci with variation between the HS progenitor strains in this study so the approach taken was to test a number of SNPs from a range of candidate biological pathways. Out of all of our SNPs, 94 were in HWE, and all of these were used for the association analysis. The remaining ones were considered only if the Chi^2^ significance was just below the 0.05 cut-off value and there was a sufficient (>3) number of minor allele homozygous animals. There were 10 of such SNPs, totalling 104 SNPs considered in our associations.

### Behavioural battery

All mice were run through a test battery designed to assess locomotor activity, anxiety and cognitive phenotypes, as previously described^42^ (Table 3).

### Statistical analysis

All analyses were conducted using the statistical software package R 2.15.2 (http://www.r-project.com/). For all analysed SNPs it was verified whether these were in HWE using a Chi^2^-test. Normality of the distribution of the investigated phenotypes was assessed using the Shapiro-Wilk test, and visual inspection of relevant histograms.

Most phenotype variables were approximately normally distributed, and for all of these, a Box-Cox transformation was applied to ensure appropriateness of applying linear models in our association analyses (with linear regression used for these analyses being nevertheless robust for data that mildly deviate from normality). Plots of the residuals (normality and residuals vs. observed values) were inspected to verify success of the transformation (Fig. S1). The only measures to which the Box-Cox transform was not applied were the ones that in our battery were found to have a censored distribution. These were mainly measures of latencies, as some animals did not perform the task in the allocated test time. These measures were analysed using the Cox proportional hazards model^47^. Morris Water Maze measures (DiffHidden1 and 5, Diffrev1 and 2, Inconsistency) representing differences between latencies to find the platform measured on different occasions were normally distributed, and these were analysed with a linear model after the Box-Cox transform.

Age and body weight at the time of killing were shown to have had a significant effect on brain weight with body weight having the largest effect, explaining approximately 17.4% of the variance in brain weight^48^. In our sample, these tended to cluster in batches, therefore analysis of variance tests were run to determine the effects of test batch on brain and behavioural phenotypes in our sample. Our results suggested that these effects were significant, therefore the batch (1–9) in which the animals arrived at the Centre was used as a categorical covariate in all association tests.

For genetic association analyses, correction for multiple testing was implemented using the Benjamini–Hochberg step-up procedure for False Discovery Rate (FDR^49^ with cut-off values q = 0.05 and q = 0.01). Measures from each behavioural test were treated as a single hypothesis, due to the high correlation between them, as indicated by the Spearman test (chosen due to mild deviation from normality in pre-transformed data); the only exceptions were the Morris Water Maze measures, which were uncorrelated, and thus treated as separate hypotheses. Each anatomical measure (body, whole brain, hippocampal and cerebellar weight) was also considered separately. Linkage disequilibrum (D’ ≥ 0.9) readouts were used in a hierarchical clustering analysis to compute the number of independent SNP groups. This highly conservative LD threshold produced 67 independent groups (only SNPs in HWE were considered; for the dendrogram of SNP clusters see Fig. S2). There were thus 871 tests performed (67 SNP clusters × 14 phenotypes). Association p-values from the tests run using Box-Cox transforms and survival analysis were pooled together for the FDR analysis, rather than considered as separate families; this was done because each SNP-phenotype association was considered under one model only. For all results we report the uncorrected p-value and the q threshold at which it was significant.

In our analyses we only considered an additive genetic model as it is the most parsimonious. Although we acknowledge that dominant or recessive models could be more appropriate for some SNP-phenotype associations, we currently have no biological grounds to think this could be the case, and considering these alternatives as well would further multiply the number of tests performed.

For the SNP-phenotype associations that remained significant after controlling for multiple testing, the distribution of residuals was further examined (visual inspection of histograms and qq plots) to ensure no unaccounted-for effects were driving the highly-significant p-values. Furthermore, in order to verify these results did not arise solely due to family structure present in the data, we used mixed models estimated using maximum likelihood procedure to analyse the top SNP-phenotype associations, entering family ID as a random effect around the intercept (package lme4). Total number of SNP-phenotype pairs investigated at this stage was used in the FDR procedure.

A hundred of the most significant SNP-phenotype associations were further examined for the possibility of existence of functional clusters. After cross-tabulating the results, an omnibus Chi^2^ test was used to analyse the association between genes involved in particular biological pathways (5HT, DA, Ach, Glu, GABA, adrenergic, neuron growth/death, other) and behavioural domains (anxiety, activity and cognitive phenotypes) or brain measures.

Gene expression analysis was carried out using the oligo Bioconductor package^50^ focusing on the genes significantly associated with the phenotypic measures. Raw cel files for 265 samples were read into R and background corrected as well as normalized using the Robust Multi-array Average (rma) algorithm. Signal intensities were summarised at the transcript level. We checked whether the genes were expressed in the mouse cortex samples, using the median expression of all transcripts as a cut-off. Subsequently, association-tests between gene expression and behavioural outcomes were run using linear models. Given expression analyses was performed only on the SNP-phenotype pairs that remained significant in the genetic association testing after the FDR correction, no multiple testing correction was applied to these results, therefore only uncorrected p-values are reported.

### Online search

In order to integrate our work with the currently existing mouse genomic resources, we verified all our results in the Northport HS stock through an online resource created by the Wellcome Trust Centre for Human Genetics (http://mus.well.ox.ac.uk/mouse/HS/)^11^. The tool allows investigation of QTLs for a number of physiological and behavioural traits in mice. Given that the Northport and Boulder heterogeneous stocks only had partially overlapping founders, the results from these two lines are not directly comparable, however, their convergence limits the possibility that a significant genotype-phenotype association is just a statistical artefact. We restricted the search to 10 Mbp upstream and downstream of the variant identified in our analyses. Given the sets of phenotypes available for our sample and Northport stock differed somewhat, for all the non-overlapping ones we identified another, best corresponding phenotype in the Wellcome Trust resource.

Furthermore, we explored the genetic variation among the inbred lines used as Boulder stock founders, using results of the Mouse Genome Project (http://www.sanger.ac.uk/science/data/mouse-genomes-project). Where further distinctions in strain nomenclature were introduced since when Boulder HS stock was started, we used the most closely related one, with our final choices being: A/J, AKR/J, BALB/cJ, C3H/HeJ, C56BL/6J, C57BL/10J, DBA/2J, I/LnJ, IS/CamRkJ, RIIIS/J (NB we looked at ten, rather than eight inbred strains, reflecting divergence of certain lines used in the Boulder stock). We looked at the number of SNPs in these strains, the proportion of exonic ones, and how many of them were likely polymorphic in the Boulder HS stock (which we defined as allelic ratio of at least 4:6 in the founders).

## Additional Information

**How to cite this article**: Janecka, M. *et al*. Genetic polymorphisms and their association with brain and behavioural measures in heterogeneous stock mice. *Sci. Rep.* **7**, 41204; doi: 10.1038/srep41204 (2017).

**Publisher's note:** Springer Nature remains neutral with regard to jurisdictional claims in published maps and institutional affiliations.

## Supplementary Material

Supplementary Information

## Figures and Tables

**Table 1:** SNP-behaviour associations significant after correcting for multiple testing.

Gene	SNP rs	Phenotype	p-value		Model	Behavioural domain	Function
			No-FS	FS			
*Sp2*	rs3708840	Elevated plus maze, total duration in the centre area	9.23e-7**	2.26e-6**	BCT	Anxiety/activity phenotypes	Transcription factor
*Tph1*	rs262731280	Light/dark exploration test, Time spent in the light chamber	5.16e-6**	9.03e-4*	S	Anxiety/activity phenotypes	5HT signalling
*Tph1*	rs262731280	Light/dark exploration test, number of transitions between light and dark chambers	1.07e-5**	2.18e-3	S	Anxiety/activity phenotypes	5HT signalling
*Map2*	rs13475902	Puzzle box, Plug (mean latency)	3.71e-4*	1.56e-2**	S	Cognitive phenotypes	nerve growth/death
*Htr3a*	rs50670893	Open field, total distance travelled in the outer zone	5.98e-4*	5.40e-2**	BCT	Anxiety/activity phenotypes	5HT signalling

As indicated in the model column, the tests were run either as a linear model, with Box-Cox transformed data (BCT), or, if the measures displayed censored distribution as a Cox proportional hazards survival model (S); the effects of batch were regressed out in all cases. P-values from models that did (FS) and did not (no-FS) control for the family structure are presented. **SNPs are significant after FDR correction at q = 0.01, *at q = 0.05.

**Table 2:** Number of SNPs on genes involved in particular biological pathways (nerve growth/death; synaptic transmission: 5HT, DA, Ach, GABA, Glu, Adr, other) associated with different phenotypes (anxiety/activity phenotypes, cognitive phenotypes, brain) among the 100 most significant associations.

	nerve growth/death	5HT	DA	Ach	GABA	Glu	other	Adr
Anxiety/activity phenotypes	1	20	1	7	6	12	1	2
Cognitive phenotypes	4	4	0	1	12	1	2	3
Brain	0	6	1	3	8	2	0	0

**Table 3:** Behavioural measures in the locomotor activity, exploration, anxiety and cognitive battery used for association testing in HS mice.

Test	measure	Description
**Spontaneous activity in home cage** - assessment of locomotion in a familiar (‘home’) environment, a baseline activity level	T1MSP	Mean speed upon transfer to home cage 0–10 min (cm/sec)
	TMSP	Mean speed upon transfer to home cage 0–60 min (cm/sec)
	HMSP	Mean speed in home cage after habituation period 0–60 min (cm/sec)
**Open field -** measure of anxiety behaviour (avoidance of the central, open area)	OFD	Time spent in the centre of the open field (sec)
	OFA	Peripheral activity: total distance travelled in the outer zone of the open field (cm)
	OFBOLI	Total boli deposited in the open field
**Elevated plus maze -** measure of anxiety behaviour (avoidance of the elevated, open arms)	P1OD	Total duration on the open arm (sec)
	P1CF	Total entries into the closed arms
	P1ND	Total duration in the centre area (sec)
**Light/dark exploration test -** measure of anxiety behaviour (avoidance of the light chamber)	LDT	Number of transitions between light and dark chambers
	LDLD	Time spent in the light chamber (sec)
	LDDA	Total distance travelled in the dark chamber (sec)
**Novel object exploration in the open field -** animal’s response towards the novel object placed in the open field	NOD	Total time spent exploring the novel object (sec)
**Puzzle box -** measure of animal’s problem solving skills.	Training	Time taken by the mouse to crawl through an underpass to enter the goal box on 2 consecutive occasions (sec)
	Burrow	Time taken by the mouse to burrow through the underpass obscured with sawdust (sec)
	Plug	Time taken by the mouse to remove a cardboard plug blocking the underpass (sec)
**Morris water maze** – assessment of spatial learning and memory. Learning in the test is indicated by decreasing latencies to find the hidden platform on repeated exposure to the maze on consecutive days.	DiffHidden1and5	The difference between the mean latency taken to find the platform on day 1 and 5 (sec)
	Diffrev1and2	The difference between the latency taken to find the platform in the second reversal task, minus the latency to find the platform in the first reversal task; during the reversal task the location of the platform was changed, so that the animal had to learn the new location. This measure reflects an animal’s cognitive flexibility (sec)
	Inconsistency	Mean difference between latencies (sums from all trials in a given session) on subsequent days (e.g. D1 and D2 morning) and at different times in a given day (e.g. D1 morning vs afternoon)
**Nesting –** animal’s propensity towards building nests (shelter/reproductive purposes)	Nest1	Nest weight on day 1 (g)
	Nest2	Nest weight on day 2 (g)

Tests were run according to the protocols specified by Lad *et al*.^42^.
