# Supplementary material for: Genetic polymorphisms and their association with brain and behavioural measures in heterogeneous stock mice
Source: Sci Rep. 2017 Feb 1;7:41204. doi: 10.1038/srep41204 (PMC5286500; doi:10.1038/srep41204)
Supplement: Supplementary Information [file srep41204-s1.pdf]

Genetic polymorphisms and their association with brain and behavioural measures in heterogeneous stock mice.

Magdalena Janecka[1,4], Sarah J Marzi[1], Michael J Parsons[1,2], Lin Liu[1], Jose L Paya-Cano[1], Rebecca G Smith[1], Cathy Fernandes[1*#], Leonard C Schalkwyk[1, 3*]

[1]MRC Social, Genetic and Developmental Psychiatry Centre, Institute of Psychiatry, Psychology and Neuroscience, King's College London, London, UK.

[2]Mammalian Genetics Unit, MRC Harwell, Oxfordshire, UK.

[3]School of Biological Sciences, University of Essex, Colchester, UK.

[4] Oxford Centre for Anxiety and Trauma, University of Oxford, Oxford, UK.

*These authors contributed equally to this work

[#] Correspondence to: cathy.fernandes@kcl.ac.uk

Supplemental Material

Figure captions

A.)

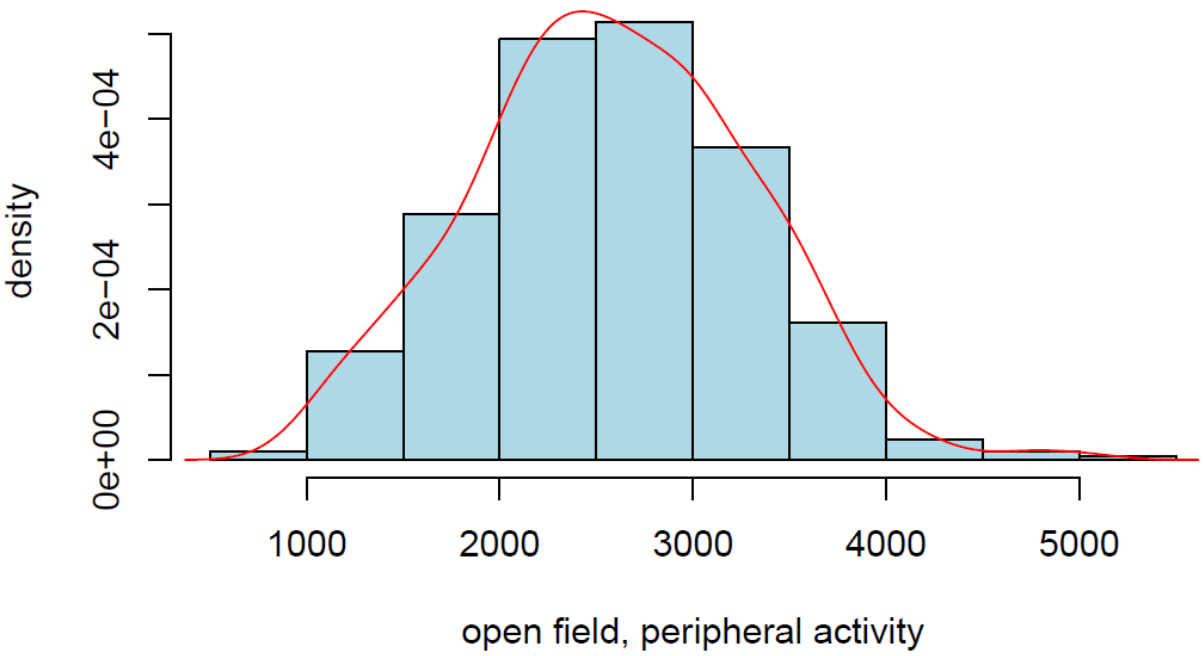

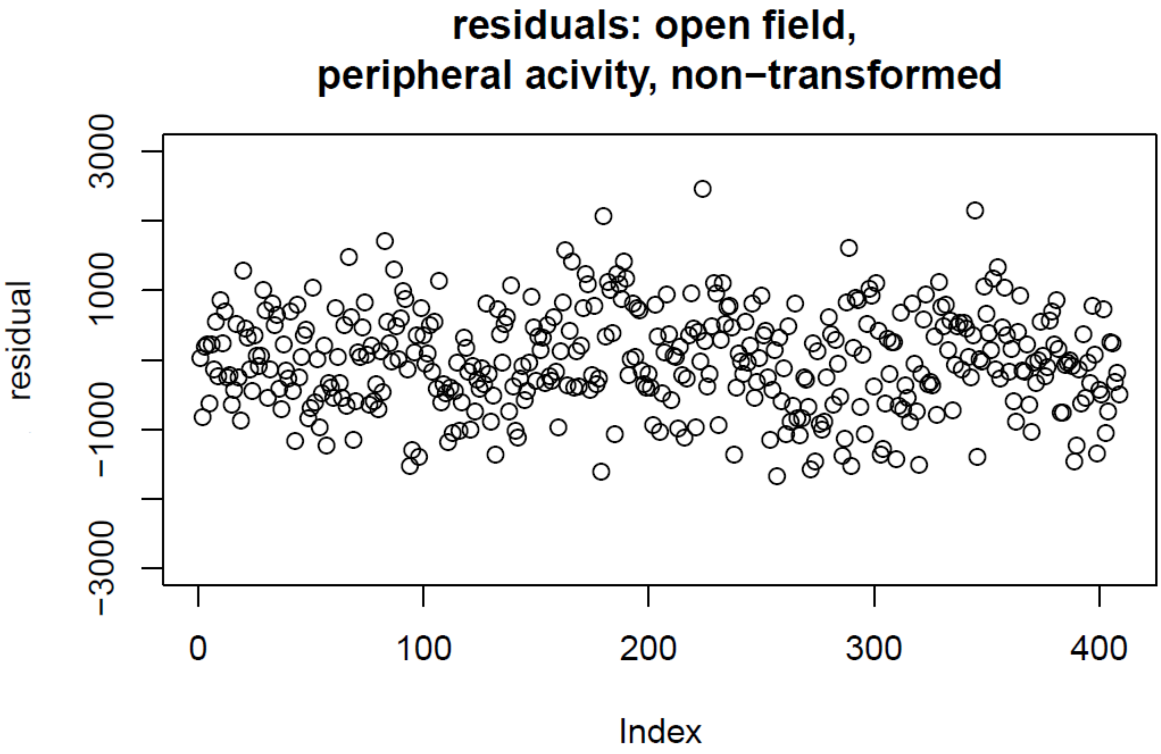

B.)

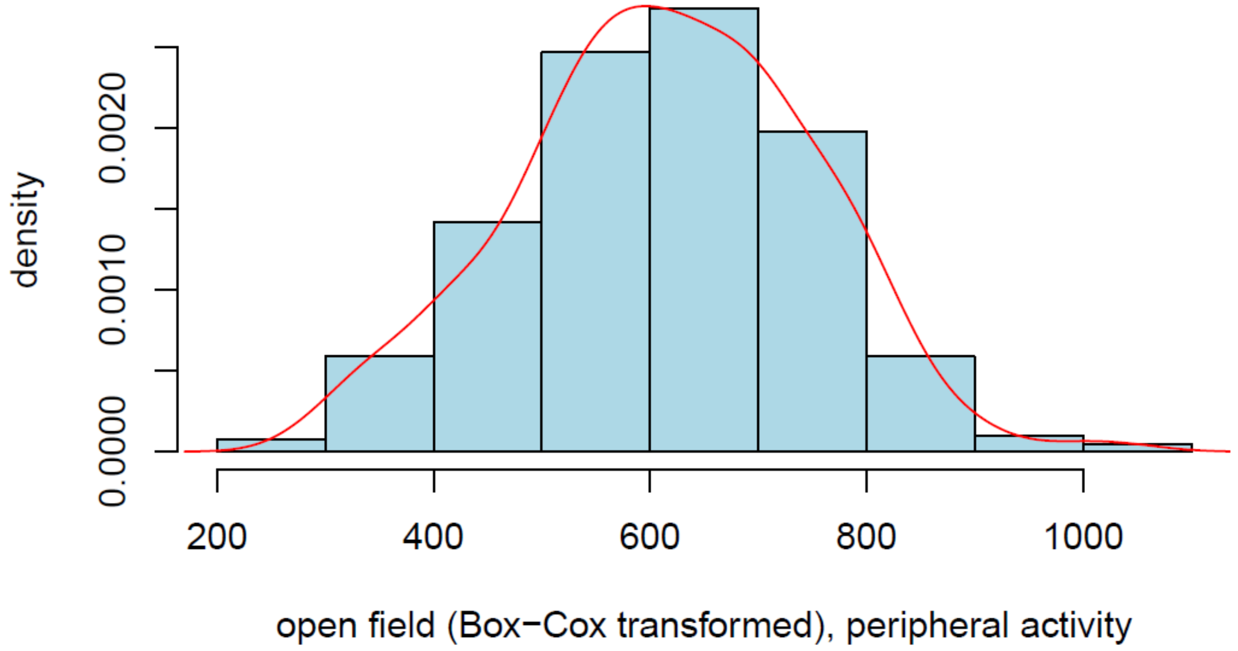

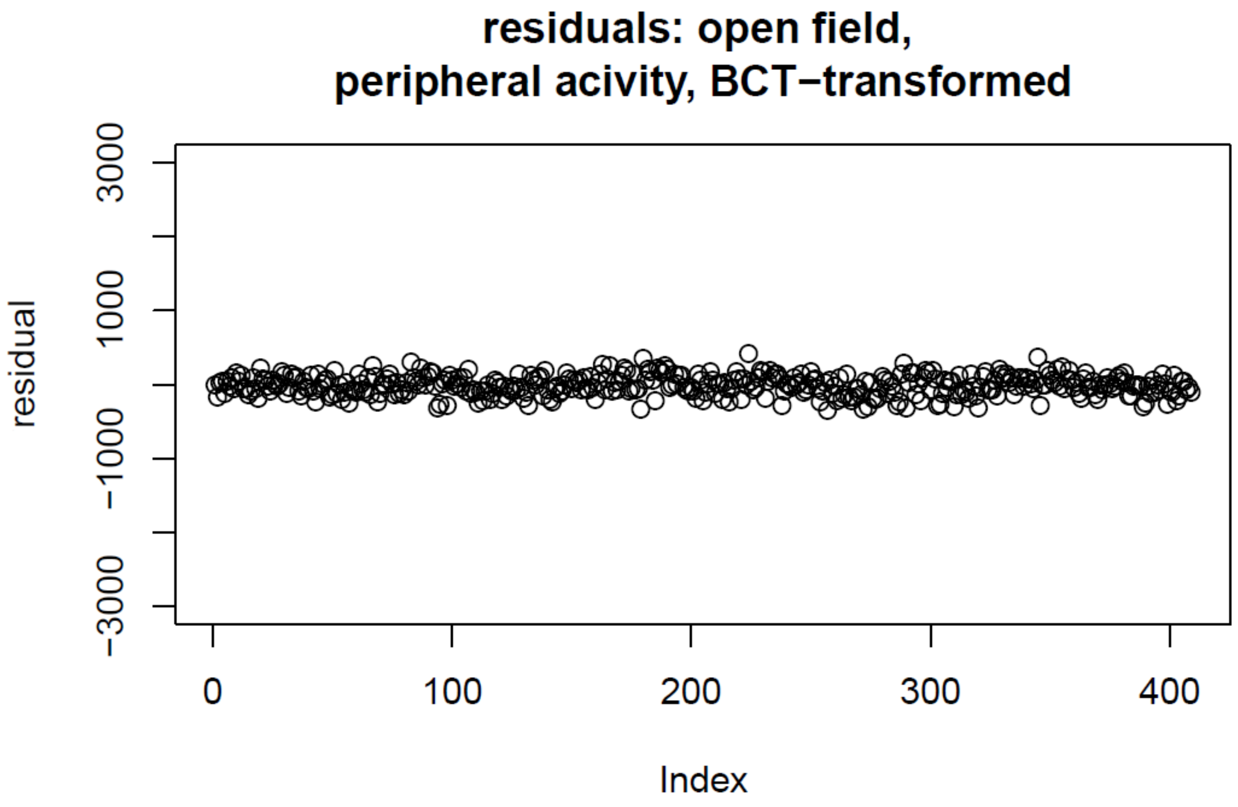

Figure S1. Normality and residuals plots before (A) and after (B) Box-Cox transformation of peripheral activity in the open field variable as an example of the transformations performed on the data. Upper panels represent normality plots; bottom panels represent residuals vs. observed values. All of the phenotypic measures that were not censored were Box-Cox transformed and analysed using a linear model.

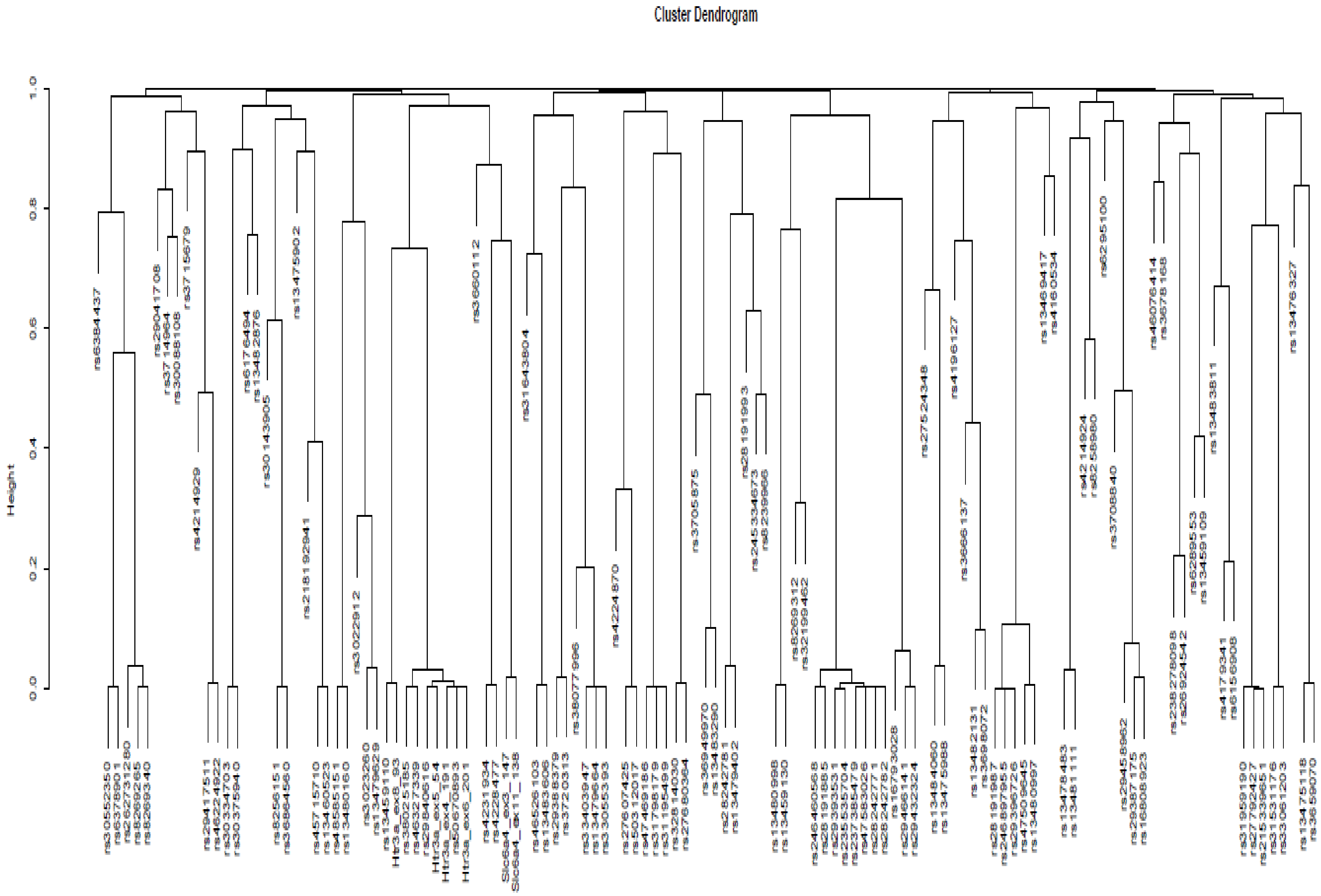

Figure S2. SNP dendrogram depicting clusters of variants in linkage disequilibrium."Height" at the top of the graph represents the D' value.

A.)

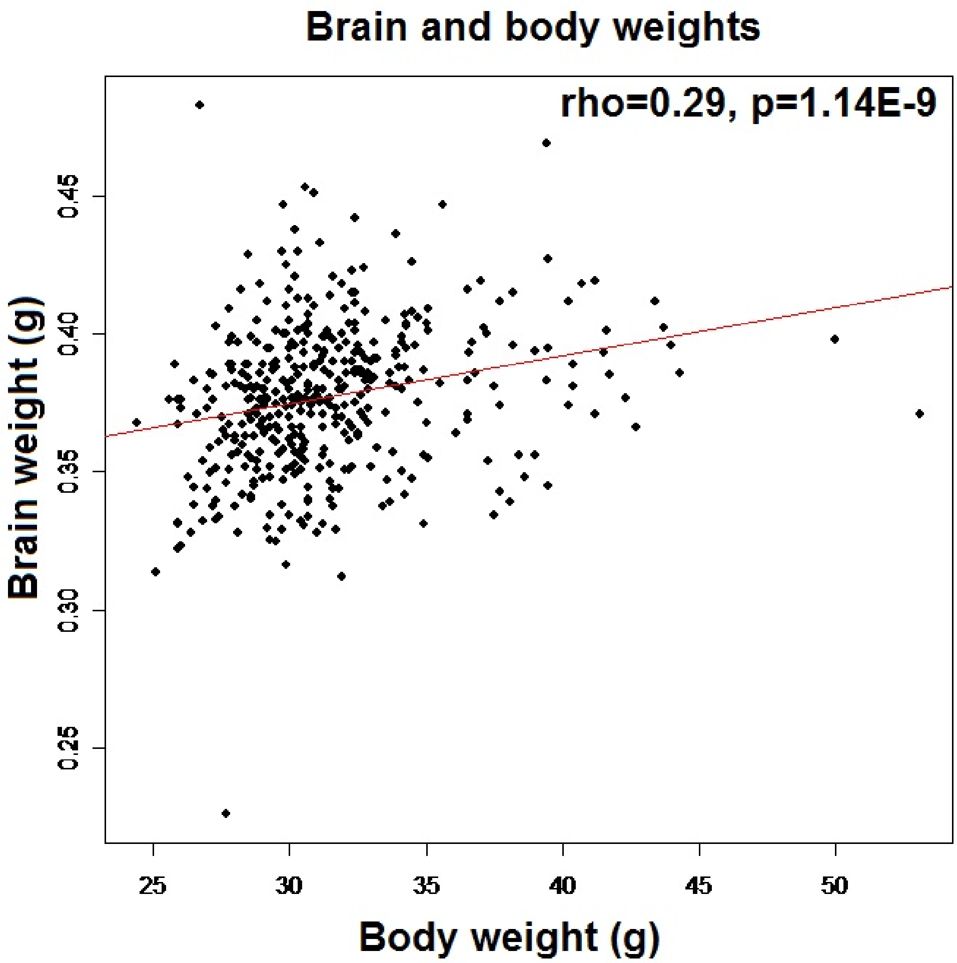
Brain and body weights
rho=0.29, p=1.14E-9
Brain weight (g)
0.25
0.30
0.35
0.40
0.45
25
30
35
40
45
50
Body weight (g)

B.)

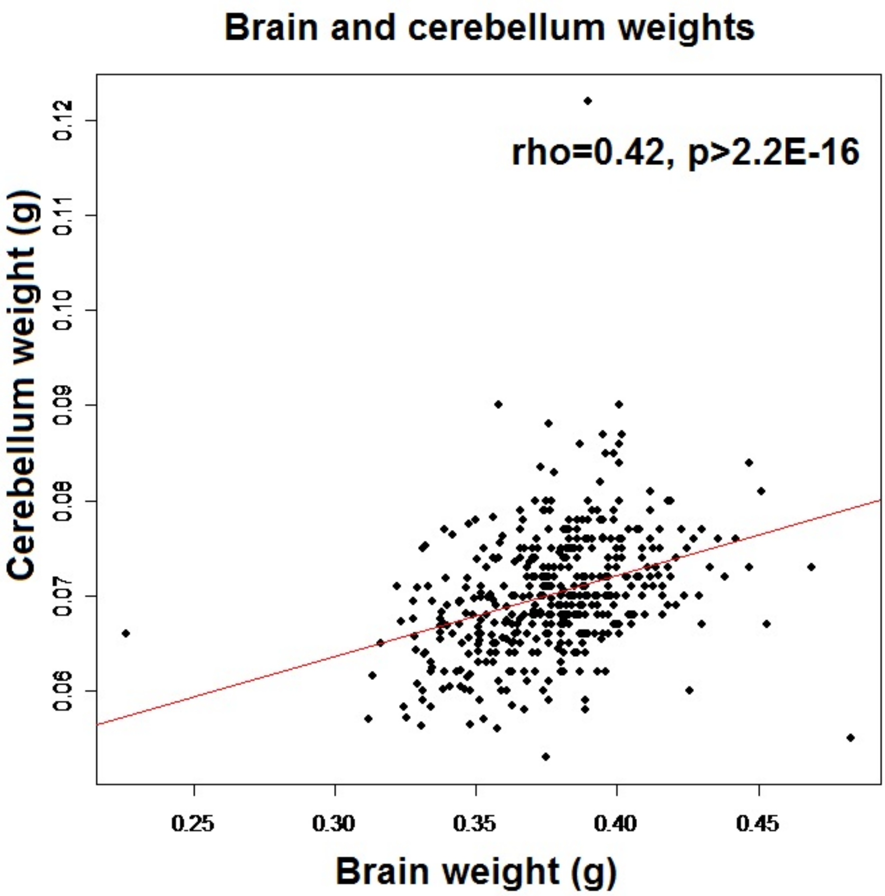
Brain and cerebellum weights
rho=0.42, p>2.2E-16
Cerebellum weight (g)
0.06
0.07
0.08
0.09
0.10
0.11
0.12
0.25
0.30
0.35
0.40
0.45
Brain weight (g)

C.)

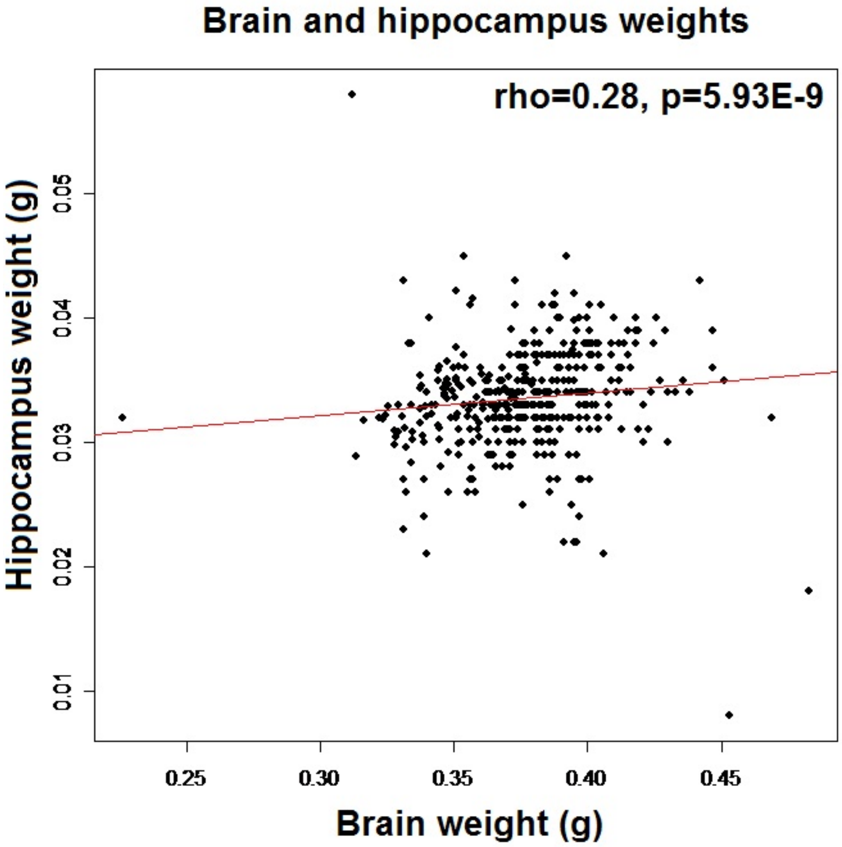

Figure S3. Correlation between brain measures in the HS mice. (A) brain – body weight, (B) brain – cerebellar weight and (C) total brain – hippocampal weight correlation.

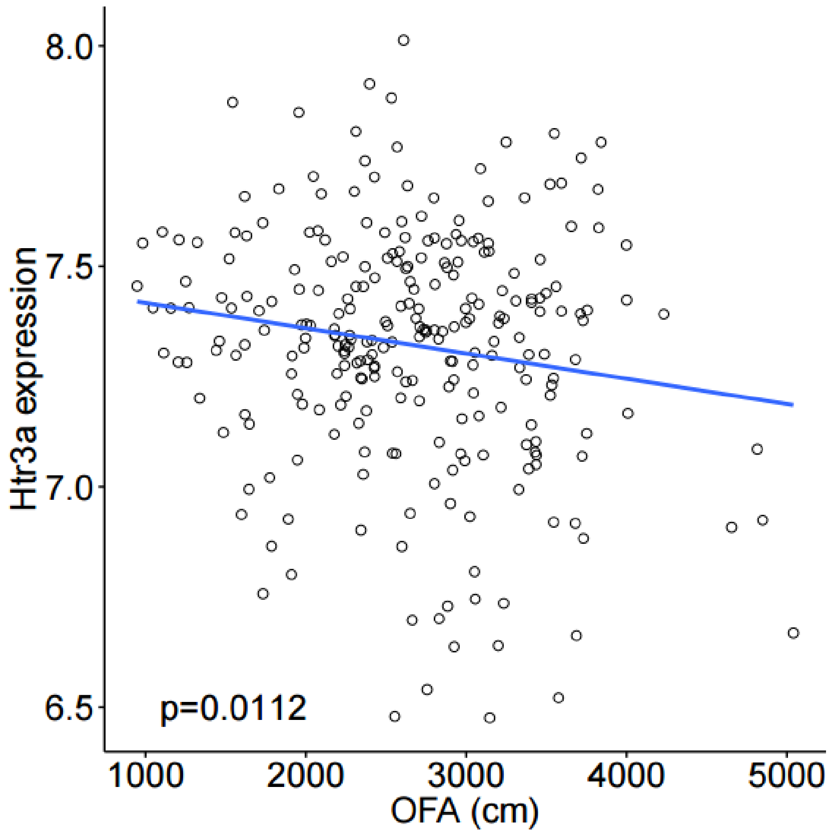

Figure S4. Association between levels of expression of *Htr3a* and distance travelled in the outer zone of the open field (OFA).

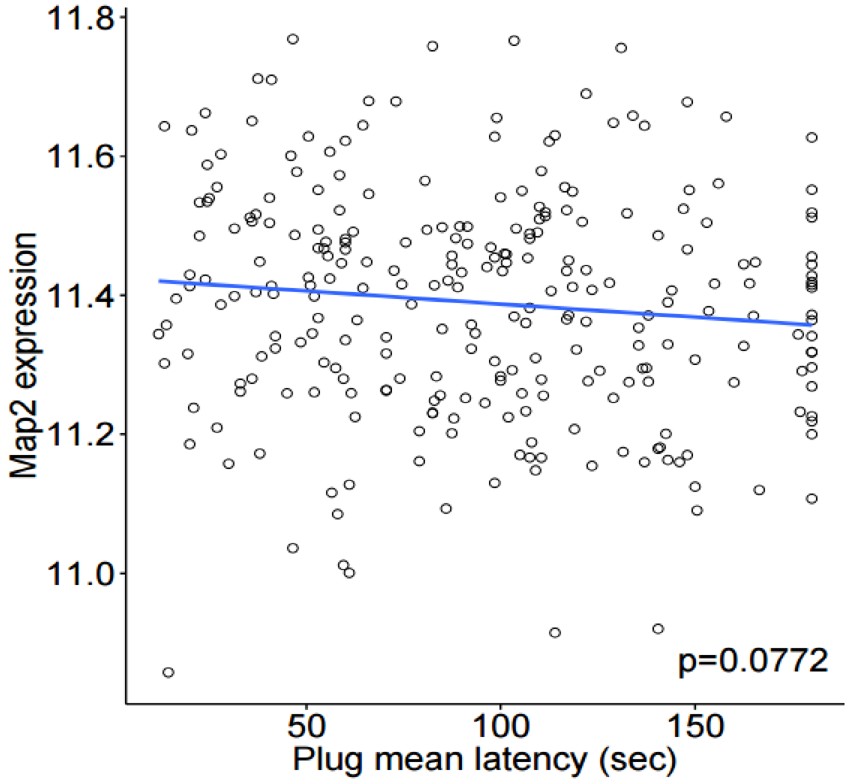

Figure S5. Association between levels of expression of *Map2* and latency to removing the plug in the puzzle box test (plug mean latency).

A.)

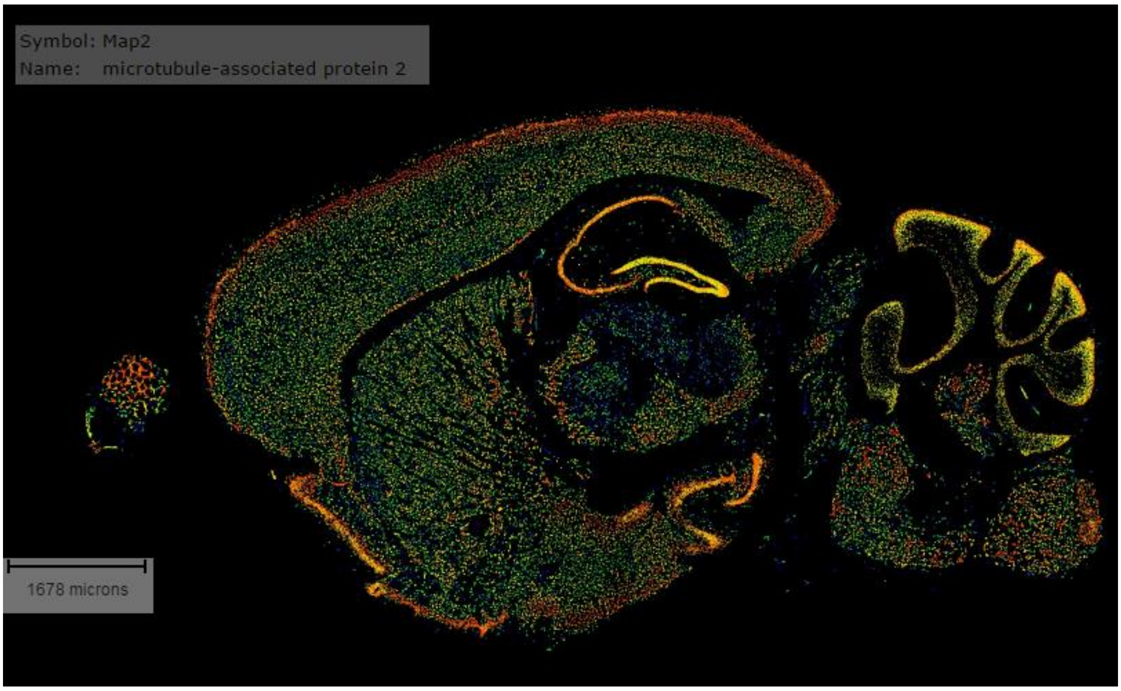

B.)

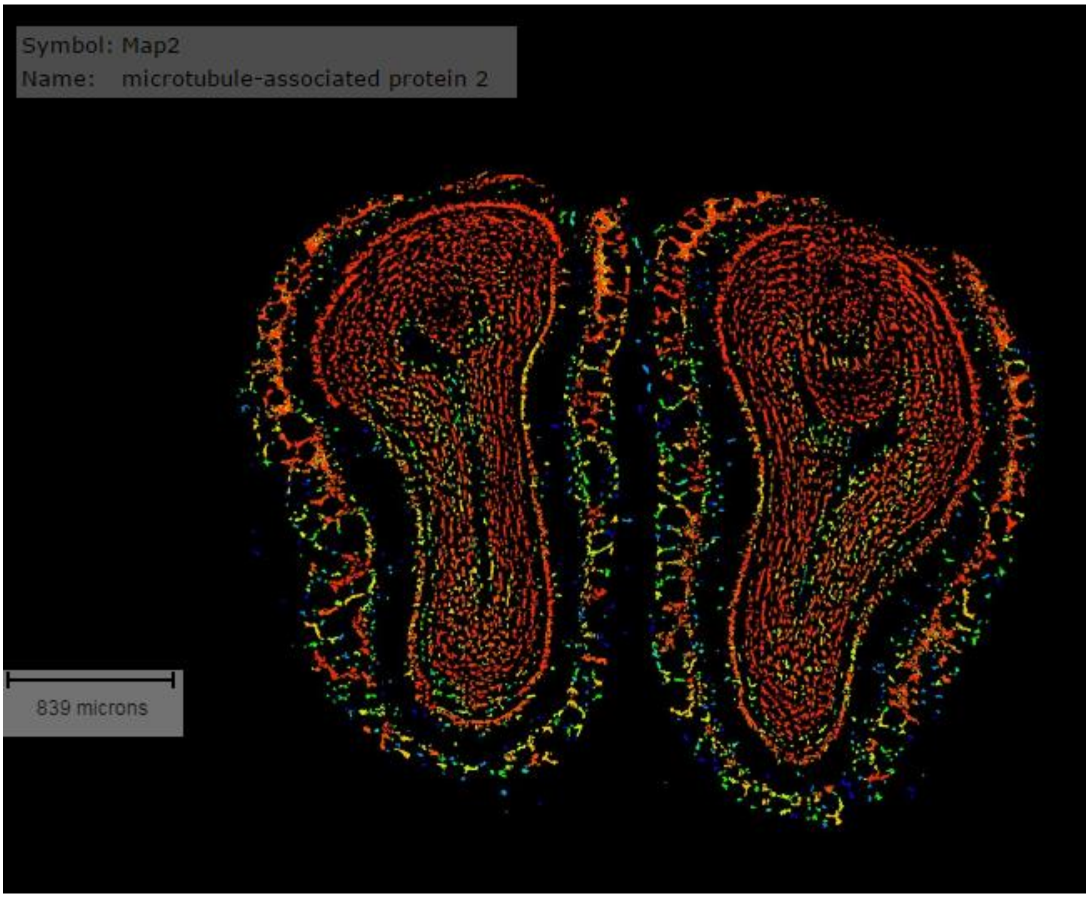

Figure S6. Map2 expression in mouse brain (C57BL/6J), A.) saggital and B.) coronal sections. Image credit: Allen Institute (Allen Mouse Brain Atlas).

Tables

Gene symbol (mouse)	gene name	SNP (rs)	allele frequency			Chi^2 p-val	location, consequence
			AA	AB	BB		
**SYNAPTIC SIGNALLING**							
**5-HT**							
**ARALKYLAMINE N-ACETYLTRANSFERASE**							
Aanat	arylalkylamine N-acetyltransferase	rs16808923	552	102	3	0.457	exon, syn.
		rs29387175	550	96	2	0.305	splice acceptor
**DOPA DECARBOXYLASE**							
Ddc	dopa decarboxylase	rs13475118	472	174	11	0.266	3' UTR
**SEROTONIN RECEPTOR**							
Htr1d	5-hydroxytryptamine (serotonin) receptor 1D	rs27607425	170	316	167	0.411	exon, syn.
		rs46526103	617	34	1	0.463	exon, syn.
		rs50312017	184	293	161	0.043	exon, syn.
Htr1f	5-hydroxytryptamine (serotonin) receptor 1F	rs4196127	146	321	186	0.737	intron

Htr2a	5-hydroxytryptamine (serotonin) receptor 2A	rs6156908	471	191	0	0.000	downstream of gene
Htr2c	5-hydroxytryptamine (serotonin) receptor 2C	rs13484060	120	0	546	0.000	intron
Htr3a	5-hydroxytryptamine (serotonin) receptor 3A	rs29980226	22	177	376	0.837	exon, syn.
		rs37082086	426	210	22	0.530	exon, syn.
		rs45878309	447	165	16	0.868	intron
		rs36586564	0	27	556	0.567	intron
		rs38025185	26	624	1	0.000	exon, syn.
		rs50670893	469	174	18	0.701	exon, syn.
		rs46327339	0	655	1	0.000	exon, syn.
Htr3b	5-hydroxytryptamine (serotonin) receptor 3B	rs29840616	422	235	1	0.000	3' UTR
Htr4	5 hydroxytryptamine (serotonin) receptor 4	rs36864560	473	172	13	0.563	exon, miss.(T/A)
		rs3705875	33	262	337	0.048	intron
Htr5a	5-hydroxytryptamine (serotonin) receptor 5A	rs3715679	157	309	160	0.750	upstream gene
Htr6	5-hydroxytryptamine (serotonin) receptor 6	rs4224870	64	256	339	0.128	downstream of gene
Htr7	5-hydroxytryptamine (serotonin) receptor 7	rs13483606	68	595	1	0.000	intron
GENES NOT TAKEN: Htr1a, Htr1b, Htr1c, Htr1e, Htr2b, Htr3c, Htr3d, Htr3e, Htr5bp							
**SOLUTE CARRIER**							
Slc25a4	solute carrier family 25 (mitochondrial carrier, adenine nucleotide translocator), member 4	rs13475988	614	1	0	0.984	exon, miss. (P/L)
Slc6a4 (SERT)	solute carrier family 6 (neurotransmitter transporter, serotonin), member 4	rs29413009	304	258	59	0.692	exon, miss.(K/R)
		rs49238038	65	233	278	0.132	exon, syn.
		rs13481111	61	258	309	0.506	3' UTR
GENES NOT TAKEN: large family of 395 genes							
**SERINE THREONINE KINASE**							
Stk31	serine threonine kinase 31	rs3698072	567	90	4	0.835	exon, miss.(S/I)
GENES NOT TAKEN:Large family of 40 genes							
**TRYPTOPHAN HYDROXYLASE**							
Tph1	tryptophan hydroxylase 1	rs32814030	646	24	0	0.637	intron
		rs262731280	572	2	0	0.967	exon, miss. (Y/C)
Tph2	tryptophan hydroxylase 2	rs4228477	540	93	2	0.338	3' UTR
**ADRENERGIC**							
**ADRENERGIC RECEPTORS**							
Adra1b	adrenergic receptor, alpha 1b	rs13480997	604	1	31	0.000	intron
		rs13480998	627	32	0	0.523	intron
Adra2a	adrenergic receptor, alpha 2a	rs3022912	467	143	12	0.785	3' UTR
Adrb2	adrenergic receptor, beta 2	rs8256151	371	255	33	0.198	exon, syn.
GENES NOT TAKEN: Adra1a, Adrra1d, Adra2b, Adra2c, Adrb1, Adrb3							
**CORTIOCOTROPIN RELEASING HORMONE RECEPTOR**							
Crhr1	corticotropin releasing hormone receptor 1	rs6384437	232	306	92	0.583	intron
GENES NOT TAKEN: Crhr2							
**CHOLINERGIC**							
CHOLINERGIC RECEPTORS							
Chrna2	cholinergic receptor, nicotinic, alpha polypeptide 2 (neuronal)	rs30552350	529	103	0	0.026	3' UTR
Chrna3	cholinergic receptor, nicotinic, alpha polypeptide 3	rs30334703	181	341	141	0.404	3' UTR
Chrna4	cholinergic receptor, nicotinic, alpha polypeptide 4	rs27680364	303	285	63	0.734	exon, miss. (T / A)
Chrna6	cholinergic receptor, nicotinic, alpha polypeptide 6	rs33403947	602	40	22	0.000	exon, miss. (S / P)
Chrnb1	cholinergic receptor, nicotinic, beta polypeptide 1 (muscle)	rs238278098	357	228	42	0.496	exon, syn.
		rs218192941	362	255	41	0.660	exon, miss.(S/A)
Chrnb2	cholinergic receptor, nicotinic, beta polypeptide 2	rs13469417	356	274	0	0.000	3' UTR

	(neuronal)						
Chrnb3	cholinergic receptor, nicotinic, beta polypeptide 3	rs13479664	602	60	0	0.222	exon, syn.
		rs33055393	0	58	0	0.000	3' UTR
Chrnb4	cholinergic receptor, nicotinic, beta polypeptide 4	rs30375940	141	341	181	0.404	exon, miss. (L/P)
Chrnd	cholinergic receptor, nicotinic, delta polypeptide	rs245334673	444	180	7	0.015	exon, miss.(V/L)
		rs31531176	463	184	12	0.196	exon, syn.
Chrne	cholinergic receptor, nicotinic, epsilon polypeptide	rs26924542	62	262	304	0.615	exon, miss. (V/M)
Chrng	cholinergic receptor, nicotinic, gamma polypeptide	rs33061203	463	186	12	0.173	exon, miss.(V/I)
GENES NOT TAKEN: Chrna1, Chrna5							
**DOPAMINE**							
DOPAMINE RECEPTOR							
Drd3	dopamine receptor 3	rs4179341	490	165	1	0.001	exon, syn.
Drd4	dopamine receptor 4	rs38077996	482	162	20	0.163	3'UTR
GENES NOT TAKEN: Drd1, Drd2, Drd3, Drd5							
**PHENYLALANINE HYDROXYLASE**							
Pah	phenylalanine hydroxylase	rs45715710	510	159	3	0.011	intron
		rs13460523	491	152	4	0.033	exon, syn.
**FATTY ACID AMIDE HYDROLASE**							
Faah	fatty acid amide hydrolase	rs8239966	452	166	6	0.028	3' UTR
**GABA**							
**GABA RECEPTOR**							
Gabra1	gamma-aminobutyric acid (GABA) A receptor, subunit alpha 1	rs246460568	434	217	19	0.187	exon, syn.
		rs246897955	176	322	158	0.653	3' UTR
Gabra3	gamma-aminobutyric acid (GABA) A receptor, subunit alpha 3	rs29041708	207	1	420	0.000	3' UTR
Gabra6	gamma-aminobutyric acid (GABA) A receptor, subunit alpha 6	rs29393531	434	219	18	0.118	exon, syn.
		rs29388379	621	5	2	0.000	splice region
		rs29396726	414	197	18	0.345	intron
		rs235535704	432	210	19	0.277	intron
Gabrb2	gamma-aminobutyric acid (GABA) A receptor, subunit beta 2	rs28191993	456	190	17	0.595	exon, syn.
		rs28191885	210	319	128	0.728	exon, syn.
		rs47509645	205	302	128	0.383	exon, syn.
		rs28191987	21	199	408	0.585	intron
		rs36949970	658	1	0	0.984	exon, syn.
Gabrb3	gamma-aminobutyric acid (GABA) A receptor, subunit beta 3	rs8269265	5	114	513	0.627	intron
		rs8269312	533	107	3	0.334	exon, stop gained
		rs8269340	506	119	6	0.732	exon, syn.
Gabrg2	gamma-aminobutyric acid (GABA) A receptor, subunit gamma 2	rs237385479	431	235	0	0.000	5' UTR
		rs47583026	482	178	8	0.058	exon, miss. (A/T)
		rs28242771	8	171	483	0.095	exon, syn.
		rs28242781	0	2	659	0.969	exon, syn.
		rs28242782	483	176	8	0.068	exon, syn.
Gabrp	gamma-aminobutyric acid (GABA) A receptor, pi	rs29466141	243	310	109	0.547	exon, syn.
		rs16793028	366	238	53	0.108	3' UTR
		rs29432324	219	316	131	0.378	3' UTR
Gabrr1	gamma-aminobutyric acid (GABA) C receptor, subunit rho 1	rs27792427	305	294	64	0.573	5' UTR
		rs31959190	176	322	158	0.653	intron
Gabrr2	gamma-aminobutyric acid (GABA) C receptor, subunit rho 2	rs46076414	65	279	290	0.861	exon, syn.
		rs215339651	309	293	64	0.650	exon, syn.
GENESE NOT TAKEN: Gabra2, Gabra4, Gabra5, Gabrb1, Gabrd, Gabrg1, Gabrg3, Gabrr3							
**GLUTAMATE**							
**CALCIUM/CALMODULIN DEPENDENT PROTEIN KINASES**							
Camk1d	calcium/calmodulin-dependent protein kinase ID	rs3678168	161	294	177	0.083	intron

		rs13476327	258	304	92	0.872	intron
Camk4	calcium/calmodulin-dependent protein kinase IV	rs13483290	7	120	507	0.973	3' UTR
GENES NOT TAKEN: Camk1a, Camk1b, Camk1g, Camk2a, Camk2b, Camk2d, Camk2g, Camk3							
**DISCS, LARGE HOMOLOG-ASSOCIATED**							
Dlgap1 (Sapap1)	discs, large (Drosophila) homolog-associated protein 1	rs3660112	43	255	358	0.790	exon (non-cod)
		rs6176494	372	219	38	0.449	intron
Dlgh2	discs, large homolog 2 (Drosophila)	rs13479402	419	184	27	0.240	exon (non-cod)
Dlgh3	discs, large homolog 3 (Drosophila)	rs3714964	369	0	257	0.000	upstream gene
GENES NOT TAKEN: Dlgap2, Dlgap3, Dlgap4, Dlgap5, Dlgh1, Dlgh4, Dlgh5							
**GLUTAMATE RECEPTOR, IONOTROPIC (AMPA / DELTA / KAINATE / NMDA)**							
Gria1	glutamate receptor, ionotropic, AMPA2 (alpha 1)	rs3023260	628	1	1	0.000	intron
Gria2	glutamate receptor, ionotropic, AMPA2 (alpha 2)	rs31643804	96	338	230	0.114	exon, syn.
Gria4	glutamate receptor, ionotropic, AMPA4 (alpha 4)	rs3666137	396	232	35	0.893	exon, syn.
Grid1	glutamate receptor, ionotropic, delta 1	rs30143905	17	160	480	0.406	exon, syn.
Grik1	glutamate receptor, ionotropic, kainate 1	rs8261020	541	1	0	0.983	exon, frameshift
		rs4214929	37	260	363	0.279	intron
		rs4214924	269	295	64	0.196	intron
Grik2	glutamate receptor, ionotropic, kainate 2 (beta 2)	rs3720313	94	290	248	0.540	intron
Grik3	glutamate receptor, ionotropic, kainate 3 (gamma 2)	rs32199462	405	210	45	0.016	exon, syn.
Grik4	glutamate receptor, ionotropic, kainate 4	rs30088108	456	164	6	0.035	exon, syn.
		rs48585151	487	168	5	0.019	exon, syn.
		rs6289553	450	165	12	0.484	intron
Grin1	glutamate receptor, ionotropic, NMDA1 (zeta-1)	rs13459110	91	293	270	0.422	exon, syn.
Grin2a	glutamate receptor, ionotropic, NMDA2C (epsilon 1)	rs4160534	81	309	244	0.271	intron
Grin2c	glutamate receptor, ionotropic, NMDA2C (epsilon 3)	rs29458962	549	112	3	0.282	splice region
		rs29417511	513	103	5	0.946	exon, syn.
		rs46224922	517	105	6	0.795	splice region
Grin2d	glutamate receptor, ionotropic, NMDA2D (epsilon 4)	rs31798179	33	250	378	0.308	intron
		rs31195499	360	268	39	0.237	exon, syn.
		rs47469186	235	269	57	0.116	exon, syn.
GENES NOT TAKEN: Gria3, Grid2, Grik5, Grin2b, Grin3a, Grin3b							
**NITRIC OXIDE SYNTHASE**							
Nos1	nitric oxide synthase 1, neuronal	rs13478483	631	1	2	0.000	3' UTR
GENES NOT TAKEN:Nos2, Nos3							
**GLUTAMATE RECEPTOR INTERACTING PROTEIN**							
Grip1	glutamate receptor interacting protein 1	rs3659070	593	70	2	0.965	intron
GENES NOT TAKEN: Grip2							
**NEUROPEPTIDE Y**							
**NEUROPEPTIDE Y RECEPTOR**							
Ppyr1	Neuropeptide Y receptor Y4	rs13482131	659	1	0	0.984	exon, miss.(R/Q)
GENES NOT TAKEN: Npbwr1, Npbwr2, Npffr1, Npffr2, Npsr1, Npy1r, Npy2r, Npy3r							
**PROTEIN TYROSINE KINASE**							
Ptk2b	PTK2 protein tyrosine kinase 2 beta	rs6378901	534	99	0	0.033	exon, miss. (K/R)
GENES NOT TAKEN: large family (see Robinson, Wu and Lin, 2000 for review)							
**OTHER**							
**G PROTEIN-COUPLED RECEPTOR KINASE**							
Grk4	G protein-coupled receptor kinase 4	rs13480160	330	287	44	0.079	
GENES NOT TAKEN: Grk1, Grk2, Grk3, Grk5, Grk6, Grk7, Grk8							
**NEURONAL GROWTH / DEATH**							
BRAIN DERIVED NEUROTROPHIC FACTOR							
Bdnf	brain derived neurotrophic factor	rs27524348	474	173	0	0.000	exon, miss. (L/M)

**EPHRIN RECEPTOR**							
Epha3	ephrin receptor 3	rs48752876	280	288	99	0.080	intron
GENES NOT TAKEN: Epha1, Epha2, Epha4, Epha5, Epha6, Epha7, Epha8, Epha9, Epha10, Ephb1, Ephb2, Ephb3, Ephb4, Ephb5, Ephb6							
**INTERLEUKIN**							
Il2	interleukin 2	rs8258980	11	646	1	0.000	exon, miss.(S/P)
IL3	interleukin 3	rs13459130	86	572	0	0.000	exon, miss.(S/N)
GENES NOT TAKEN: (multiple interleukin families (1-36))							
**MICROTUBULE-ASSOCIATED PROTEIN**							
Map2	microtubule-associated protein 2	rs13475902	183	314	134	0.974	exon, miss.(G/A)
GENES NOT TAKEN: Map1a, Map1b, Map4, Mapt							
**MICROCEPHALY**							
Mcph1	microcephaly, primary autosomal recessive 1	rs13479629	6	74	550	0.055	exon, miss. (C/F)
GENES NOT TAKEN:Mcph2, Cdk5rap2, Mcph3, Cenpj							
**NEOGENIN**							
Neo1	neogenin	rs13459112	662	1	0	0.984	exon, syn.
**PARKIN**							
Park2	Parkinson disease (autosomal recessive, juvenile) 2, parkin	rs13482876	356	278	32	0.016	exon, miss.(E/Q)
**GENE EXPRESSION**							
**AF4/FMR2 FAMILY MEMBER (FRAFILE X MENTAL RETARDATION PROTEIN)**							
Fmr2	AF4/FMR2 family, member 2	rs13483811	46	0	602	0.000	exon, miss.(T/A)
GENES NOT TAKEN: Fmr1, Fmr3, Fmr4, Fmr5							
**METHYL-CpG BINDING DOMAIN PROTEIN**							
Mbd1	methyl-CpG binding domain protein 1	rs4231934	503	139	16	0.092	exon, miss.(R/I)
GENES NOT TAKEN: Mbd2, Mbd3							
**NEURONAL PAS DOMAIN PROTEIN**							
Npas1	neuronal PAS domain protein 1	rs6295100	158	305	166	0.451	exon, miss. (L/V)
GENES NOT TAKEN: Npas2, Npas3, Npas4							
**SP TRANSCRIPTION FACTOR**							
Sp2	Sp2 transcription factor	rs3708840	198	311	148	0.221	exon, miss.(G/I)
GENES NOT TAKEN: Sp1, Sp3, Sp4							
**TIMELESS INTERACTING PROTEIN**							
Tipin	timeless interacting protein	rs13459109	261	297	74	0.450	exon, miss.(C/F)

Table S1.Complete list of SNPs used in the association analysis, grouped by broad function (dark red labels), subsets, where relevant (light red), gene families (blue) and genes. Rs numbers, allelic distribution, HWE significance, location and consequence are specified for all SNPs.

	SNP frequency			
	**0**	**≥5**	**≥10**	**≥50**
Minor allele homozygotes	48	68	78	114
Heterozygotes	31	45	45	49

Table S2. Cumulative frequencies of SNPs with minor allele homozygotes and heterozygotes equal to 0, ≥5, ≥10 and ≥50.

Test	measure	N	Mean	SD	Min	Max
**Homecage**	**HMSP** (cm/sec)	353	2.2	1.8	0.2	30.0
	**TMSP** (cm/sec)	352	4.2	1.3	1.5	24.2
	**T1MSP** (cm/sec)	352	5.1	1.1	1.6	9.8

**Open field**	**OFD** (sec)	528	9.6	10.9	0.0	78.0
	**OFA** (cm)	528	2630.2	779.3	787.7	8791.8
	**OFBOLI**	543	7.5	3.4	0	21.0
**Novel object exploration test**	**NOD**	519	30.1	28.7	0	145.2
**Elevated plus maze**	**P1OD** (sec)	539	59.7	41.4	0	265.6
	**P1CF**	539	10.2	4.0	1.0	29.0
	**P1ND** (sec)	539	100.0	28.8	3.5	219.4
**Light-dark exploration test**	**LDT**	520	15.1	11.9	0	57.0
	**LDLD** (sec)	520	90.4	55.2	0	300.6
	**LDDA** (cm)	442	846.5	233.0	0	2127.4
**Puzzle box**	**Training** (sec)	441	24.0	19.4	3.5	139.0
	**Burrow** (sec)	441	64.5	39.4	9.0	180.0
	**Plug** (sec)	351	82.3	48.6	8.5	180.0
**Nesting**	**Nest1**	353	14.9	6.1	1.6	25.0
	**Nest2**	353	14.8	6.1	1.4	25.0
**Morris Water Maze**	**DiffHidden1and5**	462	6.4	7.6	-14.5	34.3
	**Diffrev1and2**	482	2.9	5.9	-14.0	23.0
	**Inconsistency**	459	19.4	10.9	3.78	70.3

Table S3. Descriptive statistics for the behaviours measured in the HS mice. (*) indicates censored measures.

	N	Mean (g)	SD (g)	Min (g)	Max (g)
**Body weight**	420	31.47	3.80	24.40	53.10
**Brain weight**	420	0.33	0.02	0.30	0.36
**Cerebellar weight**	420	0.07	0.01	0.05	0.12
**Hippocampal weight**	420	0.03	<0.01	0.01	0.06

Table S4. Descriptive statistics for body and wet brain weights in the HS mice.

	Body weight	Brain wet weight	Cerebellum wet weight	Hippocampus wet weight
**Brain weight**	0.29 (1.14E-9)	-		
**Cerebellar weight**	0.28 (3.81E-9)	0.42 (<2.2E-16)	-	
**Hippocampal weight**	-0.03 (ns)	0.28 (5.93E-9)	0.11 (0.03)	-

Table S5. Spearman's rho correlation coefficients for body and wet brain weights. All measures, apart from hippocampus and body weights, were significantly and positively correlated with each other (denotes significance at 0.01 level, * at 0.05 level).**

Gene	SNP	phenotype	q=0.05 sig	HWE Chi^2	pathway	phenotype
Sp2	rs3708840	P1ND	SIG	0.221	circadian	anxiety
Tph1	rs262731280	LDLD	SIG	0.967	5HT	anxiety
Tph1	rs262731280	LDT	SIG	0.967	5HT	anxiety
Mapt	rs13475902	plug latency	NON	0.974	nerve growth / death	cognition
Grik3	rs32199462	Hippocampus wt	NON	0.079	glutamate	brain
Htr3a	rs50670893	OFA	NON	0.701	5HT	activity
Gabrg2	rs237385479	NOD	NON	0.000	GABA	cognition
Drd3	rs4179341	P1ND	NON	0.001	dopamine	anxiety
Gria1	rs3023260	TMSP	NON	0.000	glutamate	activity
Gabra6	rs235535704	NOD	NON	0.277	GABA	cognition
Slc6a4	rs13481111	plug latency	NON	0.506	5HT	cognition
Drd3	rs4179341	brain wt	NON	0.001	dopamine	brain

Gabra6	rs29393531	NOD	NON	0.118	GABA	cognition
Gabra1	rs246460568	NOD	NON	0.187	GABA	cognition
Chrna6	rs33403947	burrow latency	NON	0.000	cholinergic	cognition
Faah	rs8239966	plug latency	NON	0.028	endocannabinoid	cognition
Slc6a4	rs13481111	LDT	NON	0.506	5HT	anxiety
Htr2a	rs6156908	P1CF	NON	0.000	5HT	anxiety
Gria1	rs3023260	burrow latency	NON	0.000	glutamate	cognition
Htr3a	rs45878309	OFA	NON	0.868	5HT	activity
Adra1b	rs13480997	P1OD	NON	0.000	adrenergic	anxiety
Gabrg2	rs28242771	NOD	NON	0.095	GABA	cognition
Gabrb2	rs28191993	NOD	NON	0.595	GABA	cognition
Grik4	rs13480160	T1MSP	NON	0.079	glutamate	activity
Htr3b	rs29840616	brain wt	NON	0.000	5HT	brain
Gria1	rs3023260	T1MSP	NON	0.000	glutamate	activity
Gabrp	rs29432324	OFA	NON	0.378	GABA	activity
Htr4	rs3705875	T1MSP	NON	0.048	5HT	activity
Adra1b	rs13480998	plug latency	NON	0.523	adrenergic	cognition
Htr4	rs3705875	TMSP	NON	0.048	5HT	activity
Gabrp	rs29466141	OFA	NON	0.547	GABA	activity
Gabrg2	rs28242782	NOD	NON	0.068	GABA	cognition
Grid1	rs30143905	T1MSP	NON	0.406	glutamate	activity
Chrnd	rs245334673	P1ND	NON	0.015	cholinergic	anxiety
Gria2	rs31643804	P1CF	NON	0.114	glutamate	anxiety
Gabrr2	rs215339651	brain wt	NON	0.650	GABA	brain
Gabrg2	rs47583026	NOD	NON	0.058	GABA	cognition
Gabra6	rs29388379	TMSP	NON	0.000	GABA	activity
Htr3a	rs45878309	TMSP	NON	0.868	5HT	activity
Chrnb3	rs33055393	cerebellum wt	NON	0.000	cholinergic	brain
Htr6	rs4224870	OFBOLI	NON	0.128	5HT	anxiety
Htr3a	rs50670893	TMSP	NON	0.701	5HT	activity
Tph2	rs4228477	HMSP	NON	0.338	5HT	activity
Grik1	rs8261020	P1OD	NON	0.983	glutamate	anxiety
Gabrb2	rs28191885	hippocampus wt	NON	0.728	GABA	brain
Grid1	rs30143905	OFA	NON	0.406	glutamate	activity
Htr3a	rs29980226	brain wt	NON	0.837	5HT	brain
Camk1d	rs13476327	body wt	NON	0.872	glutamate	brain
Gria2	rs31643804	P1OD	NON	0.114	glutamate	anxiety
Chrnb4	rs30375940	P1OD	NON	0.404	cholinergic	anxiety
Npas1	rs6295100	Diffrev1and2	NON	0.451	gene expression	cognition
Gabrb1	rs36949970	hippocampus wt	NON	0.984	GABA	brain
Htr3a	rs36586564	NOD	NON	0.567	5HT	cognition
Slc6a4	rs29413009	LDL	NON	0.692	5HT	anxiety
Htr3a	rs37082086	plug latency	NON	0.530	5HT	cognition
Gabrp	rs29432324	NOD	NON	0.378	GABA	cognition
Chrnb3	rs13479664	cerebellum wt	NON	0.222	cholinergic	brain
Gria2	rs31643804	LDLD	NON	0.114	glutamate	anxiety

Gabrg2	rs28242781	burrow latency	NON	0.969	GABA	cognition
Htr3a	rs50670893	brain wt	NON	0.701	5HT	brain
Htr3a	rs45878309	brain wt	NON	0.868	5HT	brain
Htr3a	rs38025185	brain wt	NON	0.000	5HT	brain
Tph2	rs4228477	LDLD	NON	0.338	5HT	anxiety
Adra2a	rs3022912	Diffhidden1and5	NON	0.785	adrenergic	cognition
Chrnb2	rs13469417	P1CD	NON	0.000	cholinergic	anxiety
Htr3a	rs37082086	brain wt	NON	0.530	5HT	brain
Slc6a4	rs49238038	LDLD	NON	1.000	5HT	anxiety
Adra1b	rs13480998	P1OD	NON	0.523	adrenergic	anxiety
Gabrr1	rs27792427	brain wt	NON	0.573	GABA	brain
Htr3a	rs45878309	burrow latency	NON	0.868	5HT	cognition
Gabrr2	rs215339651	hippocampus wt	NON	0.650	GABA	brain
Chrna3	rs30334703	P1ND	NON	0.404	cholinergic	anxiety
Gabrb2	rs28191987	body wt	NON	0.585	GABA	brain
Gabrr1	rs27792427	hippocampus wt	NON	0.573	GABA	brain
Park2	rs13482876	traininig latenct	NON	0.016	nerve growth / death	cognition
Tph1	rs32814030	OFBOLI	NON	0.637	5HT	anxiety
Park2	rs13482876	NOD	NON	0.016	nerve growth / death	cognition
Chrnb4	rs30375940	P1ND	NON	0.404	cholinergic	anxiety
Grin2d	rs47469186	plug latency	NON	0.116	GABA	cognition
Grin2c	rs46224922	OFA	NON	0.795	glutamate	activity
Chrna3	rs30334703	P1OD	NON	0.404	cholinergic	anxiety
Gabrg2	rs28242781	P1OD	NON	0.969	GABA	anxiety
Gabrp	rs16793028	inconsistancy	NON	0.108	GABA	cognition
Ptk2b	rs6378901	NOD	NON	0.033	nerve growth / death	cognition
Adra1b	rs13480998	Diffrev1and2	NON	0.523	adrenergic	cognition
Htr3a	rs50670893	T1MSP	NON	0.701	5HT	activity
Gabra3	rs29041708	nest1	NON	0.000	GABA	
Gabrp	rs16793028	OFA	NON	0.108	GABA	activity
Grik1	rs4214929	HMSP	NON	0.000	glutamate	activity
Dlgh3	rs3714964	LDLD	NON	0.000	glutamate	anxiety
Grin2c	rs29458962	HMSP	NON	0.282	GABA	activity
Chrnb1	rs238278098	body wt	NON	0.496	cholinergic	brain
Htr3a	rs45878309	LDDA	NON	0.868	5HT	anxiety
Chrne6	rs26924542	HMSP	NON	0.615	cholinergic	activity
Stk31	rs3698072	P1ND	NON	0.835	nerve growth / death	anxiety
Grik4	rs48585151	nest1	NON	0.019	glutamate	
Htr3a	rs46327339	TMSP	NON	0.000	5HT	activity
Htr3a	rs38025185	OFA	NON	0.000	5HT	activity
Gabrb2	rs47509645	cerebellum wt	NON	0.383	GABA	brain

Table S6. Most significant SNP-mouse phenotype associations

	P value	Effect size	SE	DF
**P1ND ~ *Sp2* expression**	0.248	11.2	9.7	261
**LDT ~ *Tph1* expression**	0.296	-3.0	2.9	262
**LDLD ~ *Tph1* expression**	0.402	-9.9	11.8	262
**OFA ~ *Htr3a* expression**	0.011	-425.9	166.7	261
**Plug – Map2 expression**	0.077	-31.43	17.7	263

Table S7. Association statistics of behavioural measures with gene expression.
